# Light Regulates the Cytokinin-Dependent Cold Stress Responses in *Arabidopsis*

**DOI:** 10.3389/fpls.2020.608711

**Published:** 2021-02-04

**Authors:** Sylva Prerostova, Martin Černý, Petre I. Dobrev, Vaclav Motyka, Lucia Hluskova, Barbara Zupkova, Alena Gaudinova, Vojtech Knirsch, Tibor Janda, Bretislav Brzobohatý, Radomira Vankova

**Affiliations:** ^1^Laboratory of Hormonal Regulations in Plants, Institute of Experimental Botany, Czech Academy of Sciences, Prague, Czechia; ^2^Department of Molecular Biology and Radiobiology, Faculty of AgriSciences, Mendel University in Brno, Brno, Czechia; ^3^Department of Plant Physiology, Agricultural Institute, Centre for Agricultural Research, Martonvasar, Hungary; ^4^CEITEC MENDELU: Central European Institute of Technology, Faculty of AgriSciences, Mendel University in Brno, Brno, Czechia

**Keywords:** acclimation, cold stress, cytokinin, cytokinin oxidase/dehydrogenase, isopentenyl transferase, karrikin, light intensity, phytohormone

## Abstract

To elucidate the effect of light intensity on the cold response (5°C; 7 days) in *Arabidopsis thaliana*, we compared the following parameters under standard light (150 μmol m^–2^ s^–1^), low light (20 μmol m^–2^ s^–1^), and dark conditions: membrane damage, photosynthetic parameters, cytokinin oxidase/dehydrogenase (CKX) activity, phytohormone levels, and transcription of selected stress- and hormone-related genes and proteome. The impact of cytokinins (CKs), hormones directly interacting with the light signaling pathway, on cold responses was evaluated using transformants overexpressing CK biosynthetic gene isopentenyl transferase (*DEX:IPT*) or CK degradation gene *HvCKX2* (*DEX:CKX*) under a dexamethasone-inducible promoter. In wild-type plants, cold treatment under light conditions caused down-regulation of CKs (in shoots) and auxin, while abscisic acid (ABA), jasmonates, and salicylic acid (SA) were up-regulated, especially under low light. Cold treatment in the dark strongly suppressed all phytohormones, except ABA. *DEX:IPT* plants showed enhanced stress tolerance associated with elevated CK and SA levels in shoots and auxin in apices. Contrarily, *DEX:CKX* plants had weaker stress tolerance accompanied by lowered levels of CKs and auxins. Nevertheless, cold substantially diminished the impact from the inserted genes. Cold stress in dark minimized differences among the genotypes. Cold treatments in light strongly up-regulated stress marker genes *RD29A*, especially in roots, and *CBF1-3* in shoots. Under control conditions, their levels were higher in *DEX:CKX* plants, but after 7-day stress, *DEX:IPT* plants exhibited the highest transcription. Transcription of genes related to CK metabolism and signaling showed a tendency to re-establish, at least partially, CK homeostasis in both transformants. Up-regulation of strigolactone-related genes in apices and leaves indicated their role in suppressing shoot growth. The analysis of leaf proteome revealed over 20,000 peptides, representing 3,800 proteins and 2,212 protein families (data available via ProteomeXchange, identifier PXD020480). Cold stress induced proteins involved in ABA and jasmonate metabolism, antioxidant enzymes, and enzymes of flavonoid and glucosinolate biosynthesis. *DEX:IPT* plants up-regulated phospholipase D and MAP-kinase 4. Cold stress response at the proteome level was similar in all genotypes under optimal light intensity, differing significantly under low light. The data characterized the decisive effect of light–CK cross-talk in the regulation of cold stress responses.

## Introduction

Light represents one of the most important environmental signals for plants. As a source of energy, it strongly affects plant growth and development. Plants sense light via photoreceptors and rapidly adapt their gene expression and metabolism to follow changing light availability (Cejudo et al., 2019).

Plant photosystems are uniquely adaptable to low light intensities (Demmig-Adams et al., 2018). Nevertheless, low light intensity diminishes the photosynthesis rate, which is associated with delayed cell division and organ development, and slows and prolongs entire developmental programs. Low light intensity also results in the gradual spontaneous deactivation of phytochrome B due to the lack of red light (Galvao and Fankhauser, 2015), which allows up-regulation of phytochrome-interacting factors (PIFs). These factors modulate auxin biosynthesis, transport, and signal transduction (de Wit et al., 2014). Simultaneously, gibberellins (GA), brassinosteroids, and ethylene are elevated under low light conditions. In leaves, auxin stimulates the expression of the main cytokinin (CK) degrading enzyme – cytokinin oxidase/dehydrogenase (CKX), which leads to down-regulation of CK levels (de Wit et al., 2014).

Low light intensity has usually been studied in combination with shading by neighboring plants (Ballare and Pierik, 2017). Apart from the reduction in overall light intensity, neighboring plants also reflect the far-red, thus reducing the red/far-red light ratio. This reduction accelerates a decrease of the active form of phytochrome B, leading to the subsequent activation of PIFs. The shade avoidance response stimulates the elongation of hypocotyl, internodes, and petioles as well as suppression of leaf growth (Yang and Li, 2017). Prolonged shade exposure leads to diminished branching, probably by elevating abscisic acid (ABA). The response to a low red/far-red light ratio, however, is distinguishable from the general effect of low light intensity (Yang and Li, 2017).

Energy deprivation during dark treatment arrests the growth of meristematic tissues and up-regulates starvation genes, especially in the shoot apex (Mohammed et al., 2018). Prolonged dark exposure results in G_1_ being arrested irrespective of the sugar content. The auxin response is relatively high in shoot apices (Ulmasov et al., 1997), but its transporter PIN1 (pin-formed 1), is internalized, which negatively affects auxin transport toward the tips of leaf primordia and rib meristem, so preventing the formation of auxin maxima (Yoshida et al., 2011). Ethylene signal transduction [via transcription factor EIN3 (ethylene-insensitive 3)] is consistently elevated in the dark. The expression of several photosynthesis-related genes is down-regulated, e.g., marker genes for chloroplast biogenesis [GC1 (giant chloroplast 1) and ARC5 (accumulation and replication of chloroplast 5)] and vasculature-related genes. Photomorphogenesis-related transcripts, including transcription factor HY5 (elongated hypocotyl 5), are targeted by DET1 (de-etiolated 1) and COP1 (constitutive photomorphogenic 1) for degradation. Enzyme activities are affected by the redox state, the reduced forms being active in light, while inactivation by oxidation occurs in the dark (Cejudo et al., 2019). Enzyme reduction can be catalyzed in chloroplasts by ferredoxin-dependent thioredoxin reductases, which utilize the photosynthetic electron transport chain for reducing equivalents. Their function is associated with light. In contrast, NADPH-dependent thioredoxin reductases can use NADPH as a source of reducing power. In chloroplasts, some reductases have a joint domain (NtrC).

Cytokinins are among the plant hormones that have a close, positive link to light. They may have similar effects to light on plant growth and development (Miller et al., 1956). CKs can promote photomorphogenesis even in the absence of light, inhibiting chlorophyll degradation in the dark and stimulating chloroplast differentiation (Chory et al., 1994). CKs may up-regulate the expression of light-related genes in the absence of light (Cortleven and Schmulling, 2015). The CK signaling pathway also has an important function in the dark, when it maintains the levels of transcripts of several plastid genes, which enable plants to respond quickly to subsequent light (Doroshenko et al., 2016). However, the lack of hormone activation of plastid-encoded genes results in suppression of the chloroplast protein synthesis in the dark.

A sufficient light intensity is required for effective acclimation to cold stress (Szalai et al., 2009; Janda et al., 2014). Energy is necessary for activation of defense and synthesis of protective substances, especially polyamines and dehydrins. Hormonal signals may also be involved in regulation of the cold acclimation processes, as demonstrated in wheat plants (Majlath et al., 2012). However, the exact mechanisms are still poorly understood. The main questions addressed by the present study, using *Arabidopsis thaliana* as the model, are as follows: (i) How dependent on light intensity is the cold stress response? (ii) What is the role of CKs in cold stress responses? (iii) Is it possible to affect the cold stress response in different light conditions by modulating the endogenous CK content?

To answer these questions, we examined the effect of optimal, low, and absent light intensity on membrane damage, photosynthetic activity, hormone levels, proteome, and the transcription profiles of selected stress- and hormone-related genes, using *Arabidopsis* plants. The impact of CK levels on the cold response was evaluated by comparing the reaction of wild-type (WT) plants to those of transformants with either enhanced CK levels [achieved by expression of the CK biosynthetic gene *IPT* (*isopentenyl transferase*) driven by the dexamethasone (DEX)-inducible promoter] or with down-regulated CK levels (using plants expressing *CKX* under the DEX-inducible promoter).

## Materials and Methods

### Experimental Setup

Transformant lines used in this study originated from *A. thaliana* ecotype Columbia (Col-0): DEX-inducible lines *CaMV35S* > GR > *ipt* expressing *ipt* from *Agrobacterium tumefaciens* (pOp^BK^-*ipt*; *DEX:IPT*; Craft et al., 2005) and *CaMV35S* > GR > *HvCKX2* expressing *HvCKX2* from *Hordeum vulgare* (*DEX:CKX*; Černý et al., 2013). Transformed and WT plants were cultivated in a climate chamber (Percival AR41-L2) at 20°C, 60% RH, 8/16 h light/dark, under the optimal light intensity of 150 μmol m^–2^ s^–1^ using an Araponics hydroponic system consisting of 1.7-L tanks filled with 1/4 Hoagland solution. The medium was aerated every 3 h and changed after 3 weeks of cultivation and at the beginning of the cold stress.

The DEX stock solution (20 mM) was prepared in dimethyl sulfoxide (DMSO). DEX (final concentration, 10 μM) or the corresponding amount of DMSO (850 μL) was added to the hydroponic solution to 26-day-old plants. In order to evaluate the impact of DMSO, some WT plants were grown in medium without DEX or DMSO. After 24 h, plants were incubated in a fresh, pre-cooled medium supplemented with DEX/DMSO. Plants were exposed for 7 days to the stress conditions described in Table 1: cold (5°C) combined with a normal light intensity of 150 μmol m^–2^ s^–1^ (C-NL), cold (5°C) with a low light intensity of 20 μmol m^–2^ s^–1^ (C-LL), or cold (5°C) and dark conditions (C-D). Control (DEX-treated as well as DMSO-treated) plants of all tested genotypes (WT, *DEX:IPT*, and *DEX:CKX*) were kept at 20°C under 150 μmol m^–2^ s^–1^.

**TABLE 1:** The specification of experimental conditions and the abbreviations of the respective experimental variants.

Abbreviation	Conditions	Photosynthetic photon flux density (μmol m^–2^ s^–1^)	Temperature (°C)
Control	Control conditions	150	20
C-NL	Cold at normal light intensity	150	5
C-LL	Cold at low light intensity	20	5
C-D	Cold at dark	0	5

*Four-week-old plants were exposed for 7 days to the treatments.*

Fresh mass of shoots and roots of all experimental variants is shown in Supplementary Figure 1. Data were obtained from the total mass of ca 60 plants grown in two hydroponics vessels within one experiment, divided by the number of collected plants. Three independent biological experiments were analyzed (*n* = 3).

Samples of developed leaves, roots, and the shoot apical meristem with the four youngest leaf primordia (apex) were collected, frozen in liquid nitrogen, and stored at −80°C. The three independent biological experiments were performed, and number of biological replicates is specified for each analysis.

### Lipid Peroxidation

Lipid peroxidation was determined in frozen leaves as malondialdehyde (MDA) content by the thiobarbituric acid reactive substances (TBARS) method. The MDA level was used as a general marker of oxidative damage to cell membranes. The spectrophotometric measurement was performed at 532 nm, and the absorbance of interfering compounds was taken into consideration (Hodges et al., 1999). The calculation was done according to Landi (2017).

### Chlorophyll Fluorescence

Developed leaves were cut and dark adapted for 15 min at the same temperature as the respective experimental variant. After adaptation, chlorophyll fluorescence was measured using a Handy FluorCam FC 1000-H (PSI). The Kautsky characteristic was analyzed in Pulse-Amplitude-Modulated Mode (40 s dark relaxation after the saturating pulse followed by 10 min duration of actinic light 200 μmol m^–2^ s^–1^ with 15 light flashes). The maximum quantum yield of photosystem II in the dark-adapted state (*F*_*v*_/*F*_*m*_) and the steady-state PSII quantum yield in the light (QY__*Lss*_) were calculated according to Genty et al. (1989). The steady-state non-photochemical quenching in the light (NPQ__*Lss*_) was measured according to Horton and Ruban (1992).

### Activity of CK Oxidase/Dehydrogenase Enzyme

The CKX was extracted from frozen developed leaves and partially purified according to Motyka and Kaminek (1992, 1994), as modified in Motyka et al. (2003). The enzyme activity was determined by *in vitro* radioisotope assays based on the conversion of tritium-labeled *N*^6^-(Δ^2^-isopentenyl)adenine (iP) (prepared by the Isotope Laboratory, Institute of Experimental Botany of the Czech Academy of Sciences, Prague, Czech Republic) to adenine. The assay mixture (final volume 50 μL) comprised 100 mM TAPS-NaOH buffer containing 75 μM 2,6-dichloroindophenol (pH 8.5), 2 μM substrate (^2^H_3_-iP), and enzyme preparation equivalent to 16.6 or 50 mg of tissue FW. After incubation (1 h at 37°C), the reaction was terminated by adding 10 μL of Na_4_EDTA (200 mM) and 120 μL of 95% (v/v) ethanol. The substrate was separated from the product of the enzyme reaction by HPLC, as described in Gaudinova et al. (2005).

### Phytohormone Analysis

Phytohormones were extracted from frozen samples (ca 10 mg FW) by 100 μl 50% acetonitrile solution in water. Isotope labeled standards (10 pmol/sample) were added to samples: ^13^C_6_-IAA, ^2^H_4_-OxIAA, and ^2^H_4_-OxIAA-GE (Cambridge Isotope Laboratories); ^2^H_4_-SA and ^2^H_2_-GA_19_ (Sigma-Aldrich); ^2^H_3_-PA and ^2^H_3_-DPA (NRC-PBI); and ^2^H_6_-ABA, ^2^H_5_-JA, ^2^H_5_-tZ, ^2^H_5_-tZR, ^2^H_5_-tZRMP, ^2^H_5_-tZ7G, ^2^H_5_-tZ9G, ^2^H_5_-tZOG, ^2^H_5_-tZROG, ^15^N_4_-cZ, ^2^H_3_-DZ, ^2^H_3_-DZR, ^2^H_3_-DZ9G, ^2^H_3_-DZRMP, ^2^H_7_-DZOG, ^2^H_6_-iP, ^2^H_6_-iPR, ^2^H_6_-iP7G, ^2^H_6_-iP9G, and ^2^H_6_-iPRMP (Olchemim). Samples were homogenized with zirconia beads (1.5 mm diameter) in FastPrep-24^TM^ 5G Instrument (MP Biomedicals) for 40 s at 6 m/s. The extracts were centrifuged at 4°C, 30,000 *g*. The supernatant was applied to the SPE Oasis HLB 96-well column plate (10 mg/well; Waters) activated with 100 μL of methanol and eluted with 100 μL of 50% acetonitrile using a Pressure+ 96 manifold (Biotage). The sediment was re-extracted in 100 μL of 50% acetonitrile, centrifuged, and applied again to the column plate.

Phytohormones from the eluate were separated on a Kinetex EVO C_18_ column (2.6 μm, 150 × 2.1 mm, Phenomenex). Mobile phases consisted of A – 5 mM ammonium acetate and 2 μM medronic acid in water, and B – 95:5 acetonitrile:water (v/v). The following gradient program was applied: 5% B in 0 min, 7% B in 0.1 to 5 min, 10 to 35% in 5.1 to 12 min, 100% B in 13 to 14 min, and 5% B in 14.1 min. Hormone analysis was performed with a LC/MS system consisting of UHPLC 1290 Infinity II (Agilent) coupled to a 6495 Triple Quadrupole Mass Spectrometer (Agilent). MS analysis was done in MRM mode, using the isotope dilution method. Data acquisition and processing were performed with Mass Hunter software B.08 (Agilent).

### RT-qPCR

Frozen samples (up to 100 mg) were homogenized with zirconia beads in a cooled ball mill MM301 (Retsch) for 150 s at 25 Hz. RNA was isolated using RNeasy Plant Mini Kit (Qiagen). Samples were DNased using DNase I recombinant (Roche Applied Science). Total mRNA was converted to complementary DNA (cDNA) using M-MLV Reverse Transcriptase (RNase H Minus, Point Mutant, Promega), oligo-dT primers, and Protector RNase Inhibitor (Roche Applied Science). Final cDNA was diluted 20-fold with RNase-free water, and 2.5 μL of the solution was mixed with 5 μL of GoTaq qPCR Master Mix (Promega) and specific primers (see Supplementary Table 1) to a final volume of 10 μl. The PCR program (primer denaturation: 10 s at 95°C; annealing and elongation: 30 s at 60°C) was performed by Light Cycler 480 (Roche Applied Science). Ubiquitin UBQ10 was selected as the reference gene with stable transcription in all treatments, genotypes, and tissues, which is in accordance with the Genevestigator database (Hruz et al., 2008). The relative content of RNA was calculated by the ddCt method (Livak and Schmittgen, 2001).

### Proteomic Analysis

Proteins were analyzed in mixed leaf samples in three independent biological replicates. Total protein extracts were prepared as described previously (e.g., Hloušková et al., 2019). Digested and desalted peptides were analyzed by nanoflow C_18_ chromatography using a 15-cm Zorbax nanocolumn (0.1 mm inner diameter; Agilent) and a Dionex Ultimate 3000 RSLC nano-UHPLC system (Thermo). The LC was directly coupled to the Nanospray Flex (1,700 V, ion transfer tube temperature 250°C) and the Orbitrap Fusion Lumos Tribrid Mass Spectrometer (Thermo). Peptides were eluted with a 60-min, 4 to 40% acetonitrile gradient. Spectra were acquired using the default settings for peptide identification in data-dependent mode with a cycle time of 3 s. MS: resolution 60,000, scan range 375–1,500 m/z, maximum allowed injection time 50 ms, automatic gain control 4e5, 1 microscan; MS_2_: resolution 15,000, automatic gain control 5e4, maximum injection time 30 ms, 1 microscan; quadrupole isolation, HCD activation (30% Collision energy), the dynamic exclusion for 60 s (5 ppm tolerance). The measured spectra were recalibrated and searched against the reference Arabidopsis Araport 11 protein database by Proteome Discoverer 2.4, employing Sequest HT or MS Amanda 2.0 with the following parameters: Enzyme – trypsin, max two missed cleavage sites; Modifications – up to three dynamic modifications including Met oxidation, Asn/Gln deamidation, *N*-terminal acetylation, *N*-terminal Met-loss; MS_1_ tolerance – 5 ppm; MS_2_ tolerance – 0.02 Da (MS Amanda), 0.1 Da (Sequest). Only proteins with at least two unique peptides were considered for the quantitative analysis. The quantitative differences were determined by Minora, employing precursor ion quantification followed by normalization and a background-based *t* test. Interactions and functional clusters were evaluated by String (Szklarczyk et al., 2019). The mass spectrometry proteomics data have been deposited to the ProteomeXchange Consortium via the PRIDE (Perez-Riverol et al., 2019) partner repository with the dataset identifier PXD020480.

### Statistical Analysis

The effect of DMSO and DEX was evaluated by Student’s *t* test in the case of MDA, CKX, activity, and chlorophyll fluorescence measurement. The evaluation of proteome data was performed by statistical tests, which were generated using Instant Clue (Nolte et al., 2018), the Real Statistics Resource^1^ Pack software for MS Excel (Release 6.8; Copyright 2013–2020; Charles Zaiontz), Rapid Miner^2^ (Mierswa et al., 2006), and Proteome Discoverer. The data from other analyses were evaluated using Prism 8 (GraphPad). The independent component analyses (ICA) were performed by OriginPro 2020b (OriginLab).

## Results

The present study focused on elucidation of the impact of different light intensities on cold stress responses (Table 1). The applied stresses imposed significant effects on fresh mass of shoots and roots in WT, *DEX:IPT*, as well as *DEX:CKX* plants (Supplementary Figure 1). Under control conditions, the activation of *ipt* had negative effect on the root growth, while activation of *HvCKX2* negatively affected shoot growth and positively root growth. Under stress conditions, the impact of the inserted genes was diminished. The comparison of phenotypes after 7-day stress treatments is shown in Supplementary Figure 2.

### Lipid Peroxidation Induced by Cold Stress Was Diminished by CKs

**FIGURE 1:**
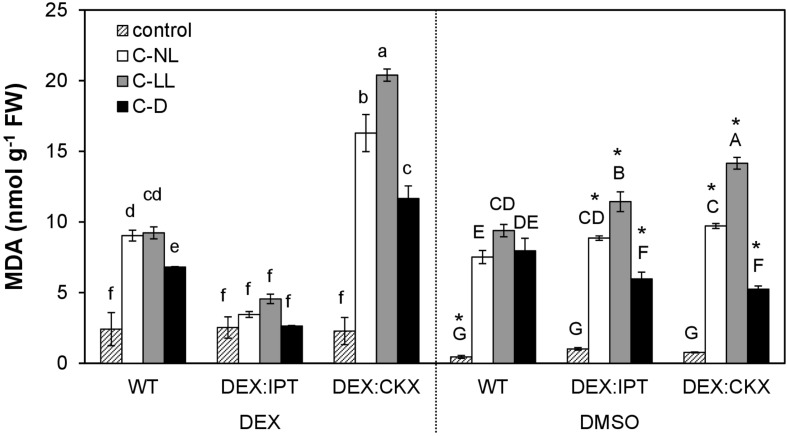
Lipid peroxidation expressed as malondialdehyde (MDA) content in leaves of wild-type (WT) and transformants *DEX:IPT* and *DEX:CKX*. See Table 1 for the description of experimental conditions. Plants were either activated with dexamethasone (DEX; diluted in DMSO) or treated with DMSO only. Means ± SD are shown. Biological samples from three independent experiments were analyzed (*n* = 3). The differences between DEX and DMSO treatments within each experimental variant were evaluated by Student’s *t* test (significant differences at *p* < 0.05 are indicated with an asterisk). The comparison among all experimental variants within the DEX (lowercase letters) or DMSO (capital letters) treatments was evaluated by one-way ANOVA with Tukey’s post hoc test (*p* < 0.05).

The stress effect of cold treatment at different light intensities was estimated as peroxidation of membrane lipids via MDA determination (Figure 1). A significant negative effect on membrane structure was observed under C-NL conditions in WT with almost a 20-fold increase in MDA. The stress impact was even more pronounced under C-LL conditions when MDA was elevated to ca 23 times that of the control. In contrast, under C-D conditions, the cold stress impact, although present, was rather lower than under C-NL and C-LL. These data are from plants treated with DMSO, but these results did not significantly differ from those of plants grown in medium without DEX or DMSO (data not shown).

The impact of CK content on stress tolerance was tested using transformants with elevated CK levels (DEX-inducible transformant with CK biosynthetic gene *ipt – DEX:IPT* plants) or with diminished CK levels (DEX-inducible transformant with gene coding for CK degradation enzyme *HvCKX2 – DEX:CKX* plants). Comparison of MDA levels in WT as well as in the non-induced transformants with plants exhibiting DEX-stimulated CK biosynthesis (*DEX:IPT*) and profound CK degradation (*DEX:CKX*) allowed evaluation of CK effect at different light intensities. DEX slightly increased the membrane damage in WT under control conditions. *DEX:IPT* transformants with activated expression of the CK biosynthetic gene showed no significant impact of cold on membrane damage. By contrast, the DEX-activated transformant with enhanced CK degradation (*DEX:CKX*) exhibited a much higher level of MDA than all the other variants. In summary, higher levels of CKs suppressed the negative effect of cold stress, while down-regulation of CK content promoted membrane damage. Membrane lipid peroxidation also depended on light intensity.

### Distinct Effect of Dark on Photosynthetic Parameters in Comparison With Light at a Wide Range of Intensities

Under the experimental conditions employed here, cold had little effect on photosynthetic parameters characterizing the photosystem II state and activity, the maximum quantum yield (*F*_*v*_/*F*_*m*_), the actual quantum yield (QY__Lss_), and the non-photochemical quenching (NPQ__*Lss*_) in steady state (Table 2). Only minor differences were observed among the tested genotypes. Mild but statistically significant inhibition of maximum quantum yield (*F*_*v*_/*F*_*m*_) was detected under C-NL conditions, especially in *DEX:IPT* plants. At the same time, the NPQ__*Lss*_ was suppressed (to similar extent in all genotypes). With the C-LL treatment, *F*_*v*_/*F*_*m*_ and QY__*Lss*_ did not generally differ from control conditions. C-D stress conditions resulted in significant down-regulation of *F*_*v*_/*F*_*m*_ and QY__*Lss*_ in all genotypes. This treatment led to the highest increase of NPQ__*Lss*_, especially in the case of *DEX:CKX* plants.

**TABLE 2:** Photosynthetic parameters.

	DEX						DMSO					
		
	WT		DEX:IPT		DEX:CKX		WT		DEX:IPT		DEX:CKX	
***F_*v*_/F_*m*_***												
Control	0.850 ± 0.001	a	0.847 ± 0.005	ab	0.838 ± 0.004	ab	0.846 ± 0.005	A	0.849 ± 0.004	A	0.847 ± 0.005	A^∗^
C-NL	0.837 ± 0.005	bc	0.823 ± 0.009	d	0.828 ± 0.005	cd	0.839 ± 0.007	A	0.821 ± 0.015	B	0.822 ± 0.008	B
C-LL	0.848 ± 0.004	ab	0.845 ± 0.005	ab	0.842 ± 0.008	ab	0.853 ± 0.005	A	0.840 ± 0.007	A	0.847 ± 0.005	A
C-D	0.820 ± 0.009	d	0.809 ± 0.007	e	0.805 ± 0.008	e	0.814 ± 0.011	B	0.810 ± 0.011	BC	0.798 ± 0.015	C
***QY*__*Lss*_**												
Control	0.521 ± 0.027	bc	0.599 ± 0.016	a	0.562 ± 0.022	ab	0.601 ± 0.009	A^∗^	0.563 ± 0.043	ABC	0.548 ± 0.056	ABC
C-NL	0.604 ± 0.026	a	0.563 ± 0.031	ab	0.578 ± 0.040	ab	0.597 ± 0.025	AB	0.523 ± 0.061	BC	0.551 ± 0.054	ABC
C-LL	0.607 ± 0.014	a	0.560 ± 0.026	ab	0.582 ± 0.008	ab	0.577 ± 0.036	ABC	0.582 ± 0.032	AB	0.591 ± 0.027	AB
C-D	0.481 ± 0.043	c	0.474 ± 0.055	c	0.466 ± 0.039	c	0.499 ± 0.071	C	0.520 ± 0.053	ABC	0.504 ± 0.050	C
***NPQ*__*Lss*_**												
Control	1.199 ± 0.188	abc	0.817 ± 0.070	cde	0.802 ± 0.117	cde	0.823 ± 0.099	BC^∗^	1.044 ± 0.186	ABC^∗^	1.113 ± 0.268	AB^∗^
C-NL	0.701 ± 0.046	e	0.611 ± 0.124	e	0.649 ± 0.114	e	0.629 ± 0.055	C^∗^	0.769 ± 0.130	BC^∗^	0.691 ± 0.119	BC
C-LL	0.785 ± 0.052	de	0.857 ± 0.154	bcde	0.750 ± 0.088	e	0.869 ± 0.136	BC	0.792 ± 0.193	BC	0.777 ± 0.087	BC
C-D	1.391 ± 0.320	a	1.162 ± 0.345	abcd	1.210 ± 0.334	ab	1.308 ± 0.541	A	0.947 ± 0.310	ABC	1.028 ± 0.241	ABC

*The maximum quantum yield of photosystem II in the dark-adapted state (*F*_*v*_/*F*_*m*_), the steady-state quantum yield of photosystem II in the light (QY__*Lss*_), and the non-photochemical quenching in steady state (NPQ__*Lss*_) of photosystem II in developed leaves of selected genotypes exposed to experimental conditions. See Table 1 for the description of experimental variants and Figure 1 for statistics. Six independent leaves were evaluated (*n* = 6).*

### Phytohormones

#### CK Down-Regulation by Cold Correlates With Diminished Light Intensity

**FIGURE 2:**
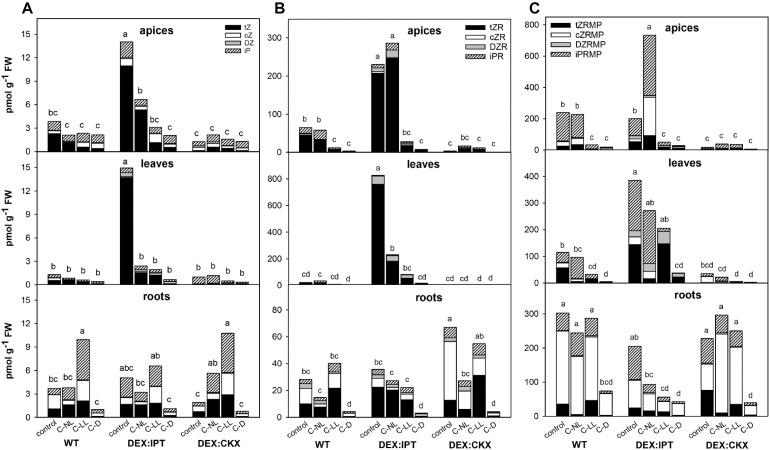
The levels of **(A)** CK bases [*trans*-zeatin (tZ), *cis*-zeatin (cZ), dihydrozeatin (DZ), and *N*^6^-(Δ^2^-isopentenyl)adenine (iP)]; **(B)** CK ribosides [*trans*-zeatin riboside (tZR), *cis*-zeatin riboside (cZR), dihydrozeatin riboside (DZR), and *N*^6^-(Δ^2^-isopentenyl)adenosine (iPR)]; and **(C)** CK phosphates [*trans*-zeatin riboside monophosphate (tZRMP), *cis*-zeatin riboside monophosphate (cZRMP), dihydrozeatin riboside monophosphate (DZRMP), and *N*^6^-(Δ^2^-isopentenyl)adenosine monophosphate (iPRMP)] in apices, leaves, and roots of wild-type (WT) and transformants with high CK synthesis after activation by dexamethasone (*DEX:IPT*), and high CK degradation after activation by dexamethasone (*DEX:CKX*) exposed to cold treatments under different light conditions (see Table 1 for the description). The expression of the transgenes was activated by dexamethasone (DEX). The statistical analyses of the total CK bases/ribosides/phosphates content were performed among all experimental variants within each tissue by one-way ANOVA with Tukey’s post hoc test in four independent biological replicates (significant differences at *p* < 0.05, *n* = 4, are indicated by different letters). Means ± SD and detailed statistics of individual CKs are shown in Supplementary Table 2.

In WT, the highly active CK *trans*-zeatin (tZ) and its riboside (tZR) were lowered in apices and leaves in the following order of treatments: C-NL > C-LL > C-D (Figure 2 and Supplementary Table 2). The content of the precursor *trans*-zeatin riboside monophosphate (tZRMP) was preserved in apices under C-NL. In contrast, the levels of tZ and tZR were slightly up-regulated in roots under C-NL and especially C-LL. Under the latter treatment, we observed iP in roots to be highly elevated. The levels of the stress-related CKs *cis-*zeatin (cZ), its riboside (cZR), and phosphate (cZRMP) were high in roots of control plants and those under C-LL. The C-D treatment resulted in low levels of all CKs in all tissues (with the exception of CK bases in apices).

Under control conditions, stimulation of the inserted genes had a profound effect on the levels of CKs. *DEX:IPT* transformant produced very high amounts of tZ, tZR, and tZRMP in apices, leaves, and, to a lower extent, roots as well (Figure 2 and Supplementary Table 2). The strong up-regulation of tZ content was partially compensated by stimulation of deactivation mechanisms such as the formation of CK *N*- and *O*-glucosides (Supplementary Table 2), as well as enhancement of CKX activity (see Figure 5 hereafter). Activated *DEX:CKX* transformant exhibited low levels of tZ in all tissues and those of tZR and tZRMP in apices and leaves (Figure 2 and Supplementary Table 2). The CK decrease caused by activated *HvCKX2* expression was compensated by an up-regulated synthesis of the precursor tZRMP and cZR in roots, by suppressed CK deactivation via glucosylation as well as by down-regulation of endogenous *CKX* expression (Figures 2, as well as see Figure 6 and Supplementary Table 2). Due to a large amount of data, only values determined in DEX-treated plants are shown.

In *DEX:IPT*, CK repression by cold was much more profound than in WT, but the levels of tZ and tZR remained under C-NL, and under C-LL in the case of leaves, higher than in WT plants grown under control conditions. C-NL led to an increase of the levels of the precursor *N*^6^-(Δ^2^-isopentenyl)adenosine monophosphate, especially in the case of apices (Figure 2 and Supplementary Table 2). The content of cZ was enhanced under C-LL in apices and roots, and that of cZRMP was enhanced only in apices (Figure 2 and Supplementary Table 2). In *DEX:CKX* plants, the C-NL treatment had a slightly positive impact on CK content (tZ, iP, and cZRMP) in comparison with control conditions; even the CK *O*-glucosylation producing storage forms was supported (Figure 2 and Supplementary Table 2). C-D treatment maintained low levels of all CKs in all tissues regardless of the genotype.

#### Auxins Were Down-Regulated by Cold Under Normal Light Conditions

**FIGURE 3:**
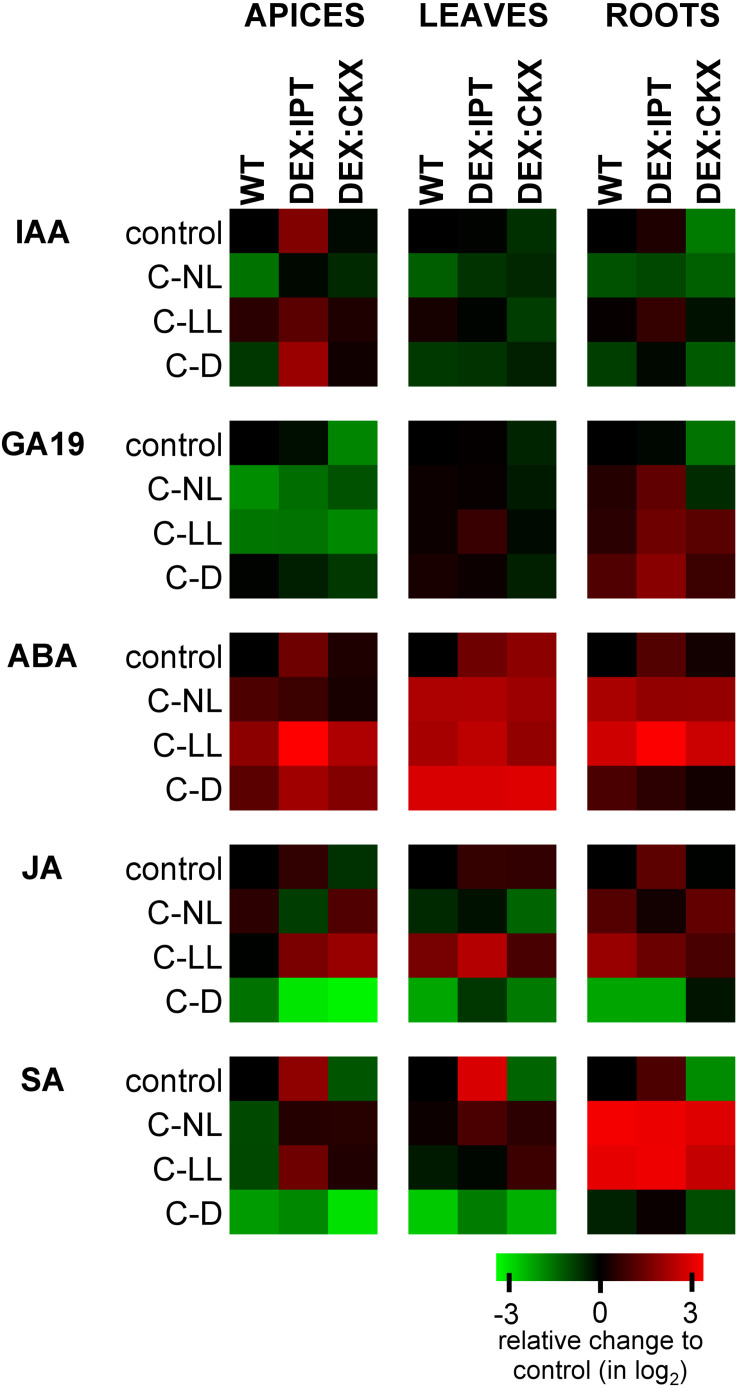
The relative levels of phytohormones in apices, leaves, and roots of WT, *DEX:IPT*, and *DEX:CKX* plants expressed as a log_2_ change of the ratio between the genotype under individual treatments and the WT under control conditions within each tissue separately. The expression of the transgenes was activated by dexamethasone (DEX). See Table 1 for the description of experimental variants. ABA, abscisic acid; SA, salicylic acid; JA, jasmonic acid; GA19, gibberellin GA_19_; IAA, auxin indole-3-acetic acid. The means ± SD and detailed statistics of hormones are shown in Supplementary Table 2.

The C-NL treatment suppressed the production of auxin indole-3-acetic acid (IAA) in WT plants (Figure 3 and Supplementary Table 2). C-LL only had a minor effect on IAA levels. In contrast, strong IAA suppression was observed under C-D conditions. Under C-NL treatment, IAA was predominantly converted to the reversible storage form (its glucose ester) in all tissues, under C-LL only in roots (Supplementary Table 2). Under C-D conditions, IAA was partially deactivated to the irreversible amino acid conjugates, IAA-aspartate and IAA-glutamate (Supplementary Table 2).

In comparison with WT, *DEX:IPT* plants exhibited high IAA elevation in apices and also, but to a lesser extent, in roots (Figure 3). In this genotype, IAA under control conditions was actively converted to its major primary catabolite 2-ox-indole-3-acetic acid in roots (Supplementary Table 2). In comparison with the other genotypes, *DEX:CKX* plants had, under control conditions, lower IAA content in leaves and especially in roots.

Cold treatments had similar effects on *DEX:IPT* plants as on WT, leading to a significant decrease of IAA under C-NL, while only a minor effect was found under C-LL. However, under C-D, relatively high IAA levels were detected in apices. *DEX:CKX* plants maintained the same IAA concentration under cold treatments as under control conditions, with the exception of a mild elevation under C-LL, especially in roots.

#### Gibberellins Were Affected Predominantly in Apices

Suppression of the GA precursor GA_19_ indicated down-regulation of GAs in the cold at two levels of illumination (C-NL and C-LL) in apices of WT as well as *DEX:IPT* (Figure 3 and Supplementary Table 2). Up-regulation of GA_19_ was detected in roots of the latter genotype in all cold treatments, especially in the dark (C-D). Under control conditions, down-regulation of CKs in *DEX:CKX* plants led to a decrease of the levels of GA_19_ in apices and leaves. During cold treatments, this genotype exhibited levels of GAs similar to those in WT.

#### ABA Was Up-Regulated by all Cold Treatments

After 7-day treatment under C-NL, only moderate ABA elevation was observed in WT apices (Figure 3 and Supplementary Table 2). In leaves and roots, ABA was significantly elevated. Under C-LL, ABA increased substantially in all WT tissues. Under C-D, ABA increased especially in leaves.

In control conditions, *DEX:IPT* plants had basal levels of ABA ca 2.5 times higher than WT in all tissues, while in *DEX:CKX* plants, this only occurred in leaves. In comparison with WT, ABA increase was prominent in all *DEX:IPT* tissues under C-LL. Under C-D, all genotypes exhibited similar high ABA up-regulation in leaves. The ABA catabolites – phaseic acid, dihydrophaseic acid, and 9-hydroxy-abscisic acid (Supplementary Table 2) – remained at low levels in apices and leaves.

#### Jasmonates Were Stimulated by Cold at Low Light Conditions

Jasmonic acid (JA) and its active isoleucine conjugate were moderately up-regulated under C-NL in WT roots and highly up-regulated under C-LL in leaves and roots (Figure 3 and Supplementary Table 2). Under C-D conditions, the contents of JA and its isoleucine conjugate were strongly suppressed in all tissues. *DEX:IPT* plants had higher JA basal levels in all tissues, while *DEX:CKX* had lower levels in apices. Under C-D, *DEX:IPT* plants exhibited a strong JA down-regulation in roots, similarly to WT.

#### Salicylic Acid Content Correlated With CKs

Both cold treatments with light caused a moderate decrease of salicylic acid (SA) levels in apices, a negligible effect in leaves, and a strong positive effect in roots (Figure 3 and Supplementary Table 2). SA content correlated well with CK levels in all organs. Under control conditions, the *DEX:IPT* genotype contained high SA levels in all tissues, while in *DEX:CKX*, they were lower. Both C-NL and C-LL treatments enhanced the synthesis of SA in roots to similar levels in all genotypes and, to a minor extent, also in *DEX:IPT* and *DEX:CKX* apices and leaves. The C-D treatment was associated with very low SA content in all tissues of all genotypes.

#### Phytohormone Changes in Summary

**FIGURE 4:**
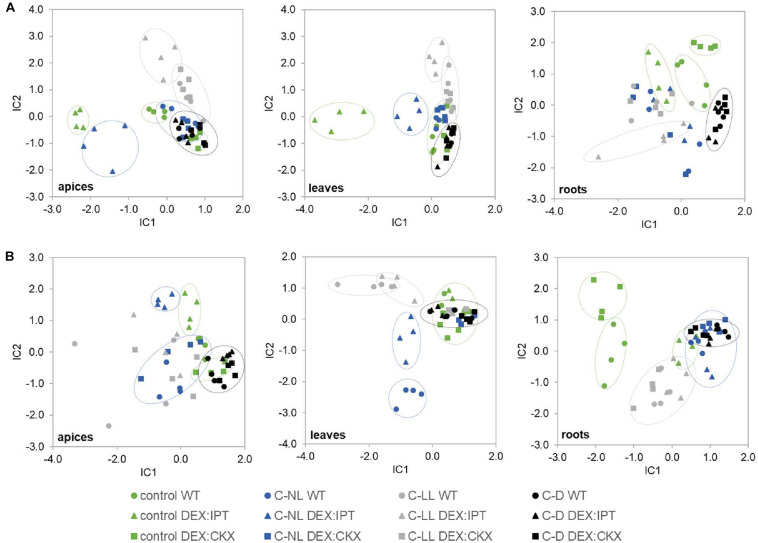
The independent component analysis (ICA) of **(A)** hormonal and **(B)** transcriptomic data in apices, leaves, and roots of WT, *DEX:IPT*, and *DEX:CKX* plants exposed to cold stress under different light conditions (see Table 1 for the description of variants). All samples are shown as individual points (four biological replicates of each variant). Groups with high diversity are highlighted by circles. In the case of transcriptomic data, the inserted genes were excluded from the analysis.

Independent component analyses of total phytohormones in apices and leaves (Figure 4A) has clearly shown the divergence of the *DEX:IPT* genotype from the other two genotypes. The response of this transformant to cold stress treatments was the most distinct from control conditions. In general, C-NL stressed plants differed from both C-LL- and C-D-treated plants at the hormonal levels in all tissues (separation on IC2 axis). C-D-treated plants showed the same behavior regardless of genotype. The *DEX:CKX* genotype exhibited a phytohormone profile similar to dark-treated plants, except in the roots, which indicates the intensive stress response of this tissue.

**FIGURE 5:**
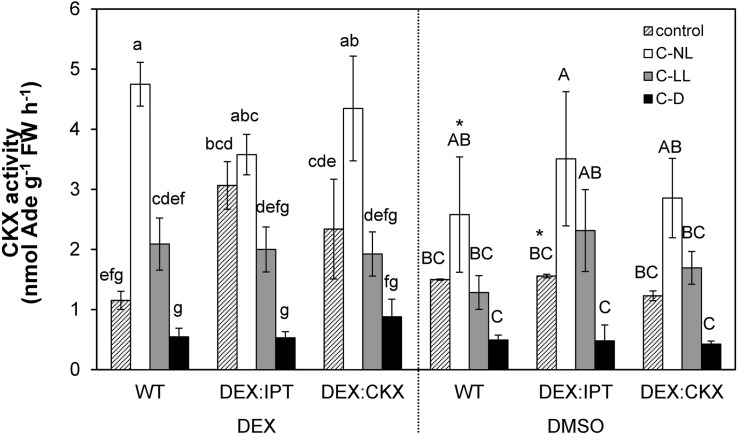
The activity of cytokinin oxidase/dehydrogenase (CKX) enzyme in leaves of WT, *DEX:IPT*, and *DEX:CKX* plants exposed to cold treatments under different light conditions. Plants were activated by dexamethasone diluted in DMSO (DEX) or treated by pure DMSO. See Table 1 for the description of experimental variants and Figure 1 for statistics (n = 3).

**FIGURE 6:**
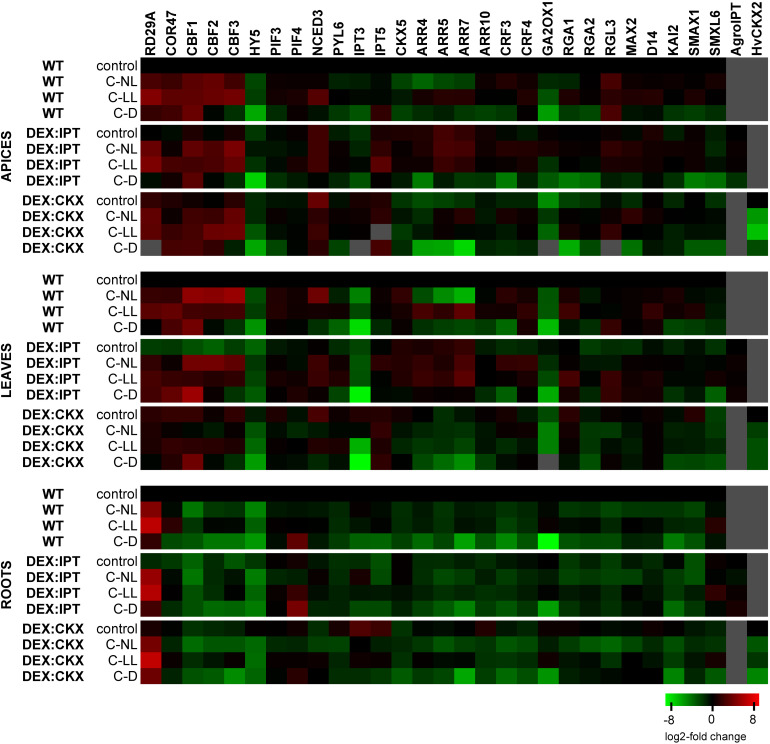
The transcription changes of selected genes related to WT in control conditions within each tissue separately. Means ± SD and detailed statistics of gene transcription are shown in Supplementary Table 3. Gray squares indicate the transcription level to be below the detection limit. The expression of the transgenes was activated by dexamethasone (DEX).

### CK Oxidase/Dehydrogenase Activity Increased in Both Transformants and Cold Treatments in Light

Under C-NL, WT exhibited a strong up-regulation of CKX activity in leaves (Figure 5). Under C-LL conditions, CKX activity was close to the control levels. Low CK content under C-D was associated with very low CKX activity.

Cytokinin oxidase/dehydrogenase activity in transformants treated with DMSO did not differ significantly from WT plants. After DEX treatment, CKX activity was substantially elevated in leaves of *DEX:IPT* plants grown in control conditions, probably due to the plant efforts to re-establish CK homeostasis. CKX activity also increased, but to a lower extent, in *DEX:CKX* plants, because of the activation of *HvCKX2* expression. Under C-NL conditions, an intense stimulation of CKX activity was detected, slightly above the levels of DEX-activated *DEX:IPT* transformant under control conditions. Under C-LL conditions CKX activity was only moderately stimulated, and to a similar degree in all genotypes. CKX activity under C-D was very low in all genotypes, although slightly higher in *DEX:CKX*.

### Gene Transcription

#### Transformant Characterization

The expression of both introduced genes (*ipt* from *A. tumefaciens* and *HvCKX2* from *H. vulgare*) was, after activation with DEX, verified by RT-qPCR (Figure 6 and Supplementary Table 3; data from DMSO treatment not shown). The transcription of *ipt* in *DEX:IPT* plants did not change with experimental conditions except in apices under C-D conditions. In contrast, the activated *DEX:CKX* plants exhibited high transcription of *HvCKX2* under control conditions, which was significantly down-regulated by all cold treatments, especially in the dark.

#### Light Intensity Affected Cold Responsiveness of Stress-Related Genes

The transcription profiles of stress-related genes are shown in Figure 6 and Supplementary Table 3. Under control conditions, the transcription of *RD29A* (*responsive to desiccation 29A*) was most profound in *DEX:CKX* plants, less so in *DEX:IPT.* However, C-NL stress up-regulated *RD29A* transcription after 7 days to the highest extent in the apices and roots of *DEX:IPT* plants. The C-LL treatment substantially elevated *RD29A* transcription, less so in *DEX:CKX* apices and leaves. A very high increase of its transcription was detected in roots of all genotypes. Under C-D, the most profound transcription was found in *DEX:IPT* leaves. The transcription of *COR47* (*cold-regulated 47*) had, under control conditions, a similar profile to that of *RD29A*. Under C-NL, the stimulation of *COR47* transcription was most profound in WT apices and leaves, while, compared with the control, it was enhanced only in roots of *DEX:IPT* where it reached the level of WT. The most substantial *COR47* up-regulation was found in the C-LL-treated plants, especially in WT plants. Under C-D, *COR47* transcription was also up-regulated, predominantly in the case of *DEX:CKX* apices and *DEX:IPT* leaves, where it was more than double that under C-LL conditions.

The key transcription factors associated with cold stress are C-repeat binding factors (CBFs). The transcription of *CBF1*, *2*, and *3* was up-regulated by all cold treatments in apices and leaves, substantially in the case of C-NL, except in *DEX:CKX* leaves. *CBF1* transcription was also considerably enhanced under C-D, especially in leaves of *DEX:IPT*. In roots, the expression was generally low.

Elongated hypocotyl 5 (HY5) is the hub, light-related transcription factor, which coordinates light and hormone signaling pathways. Under control conditions, *HY5* transcription was relatively low in *DEX:IPT* plants in comparison with other genotypes. All 7-day cold treatments (especially C-D) diminished its transcription, with the exception of apices and leaves of *DEX:IPT* plants under C-NL, when it was promoted in comparison with *DEX:IPT* control.

Low light intensity responses are associated with PIFs. In apices, *PIF3* transcription was significantly down-regulated after prolonged C-D treatment. In leaves, both C-NL and C-LL conditions resulted in the up-regulation of *PIF3* and *4* in WT and *DEX:IPT* plants. In roots, C-NL was accompanied by *PIF3* down-regulation, while *PIF4* was highly transcribed under C-D conditions, but to a lesser extent in *DEX:CKX* plants.

#### Hormone-Related Genes

The transcription changes of hormone-related genes in DEX-treated plants are shown in Figure 6 and Supplementary Table 3.

##### Cytokinins

The basal transcription of the CK biosynthetic gene, *IPT3*, was strongly suppressed in *DEX:IPT* plants, while it was highly promoted in the *DEX:CKX* transformant. Similarly, transcription of another CK biosynthetic gene, *IPT5*, was stimulated in *DEX:CKX* leaves and roots. C-NL conditions reduced *IPT3* transcription in *DEX:CKX* plants, which remained, however, higher in apices and leaves than in WT plants. By contrast, the transcription in *DEX:IPT* roots under C-NL was up-regulated compared with the control. The *IPT3* transcription was higher in apices of all genotypes under C-LL conditions than under C-NL, while under C-D, it was very low, close to the detection limit. By contrast, *IPT5* was substantially up-regulated in apices and leaves, especially in *DEX:CKX*.

Opposite to *IPTs*, transcription of the gene for the CK degradation enzyme, *CKX5*, was, under control conditions, elevated in apices and leaves of *DEX:IPT* and strongly diminished in apices and roots of *DEX:CKX*. It was also generally enhanced in *DEX:IPT* plants exposed to cold treatments. C-NL promoted *CKX5* transcription in WT leaves but diminished it in apices and roots. C-D conditions were associated with the lowest abundance of *CKX5*.

The CK signal transduction via type-B *ARR10* (*Arabidopsis response regulator 10*) was promoted in control conditions especially in roots of *DEX:CKX* plants. C-NL and C-LL diminished this up-regulation, resulting in transcription levels comparable with the other genotypes. C-D suppressed *ARR10* transcription in all tissues of all genotypes. The transcription profiles of type-A response regulators *ARR4*, *5*, and *7* were similar. Under control conditions, the expression correlated with CK levels in apices and leaves. C-NL treatment inhibited the transcription of these regulators in all tissues, except for *DEX:IPT* apices and leaves, which maintained high transcript levels. In the case of C-LL, the up-regulation of type-A response regulators was found in apices and leaves of WT and *DEX:IPT*, while it was kept low in the *DEX:CKX* transformant. C-D resulted in low transcription of these regulators, being the highest in the case of leaves of the *DEX:IPT* genotype.

The transcription levels of *CRF3* and *4* (*cytokinin response factors*) were generally lower in *DEX:CKX* plants in comparison with other genotypes. The most significant response was up-regulated transcription under C-NL in apices and leaves (although there was no response in *DEX:CKX* leaves) and down-regulation under C-D. In roots, all stress treatments were associated with *CRF3* and *4* suppression. *CRF3* exhibited a more profound response under C-NL, while *CRF4* transcription was most prevalent under C-LL.

##### Abscisic acid

The transcription level of the gene coding the rate-limiting ABA biosynthetic enzyme *NCED3* (*9-cis-epoxycarotenoid dioxygenase 3*) was highest under control conditions in apices and leaves of *DEX:CKX* plants. A mildly increased level was also detected in apices of the *DEX:IPT* transformant. The most prominent change in leaves was an increase in *NCED3* in WT under C-NL, while in *DEX:CKX* plants, *NCED3* transcription was down-regulated. In roots, C-LL and C-D treatments elevated its transcription in *DEX:CKX* plants. The transcription of the ABA receptor *PYL6* (*PYR-like 6*) was diminished in the *DEX:IPT* genotype under control conditions, but it was elevated in apices and leaves under both C-NL and C-LL conditions. Darkness repressed *PYL6* transcription since low *PYL6* levels were detected in roots under C-D stress.

##### Gibberellins

Transcription of the gene for the GA deactivating enzyme *GA2ox1* (*gibberellin 2 oxidase 1*) was low in the case of apices and leaves of *DEX:CKX* plants under control conditions. Cold treatments inhibited its transcription in these tissues in all genotypes (except C-NL treatment in *DEX:IPT* apices). In roots, *GA2ox1* was down-regulated only in the dark (C-D). Under control conditions, transcription of DELLA proteins *RGA1*, *RGA2* (*repressors of GA*), and *RGL3* (*RGA-like 3*) was much lower in apices of *DEX:CKX* plants than in the other genotypes. By contrast, *DELLAs* were less transcribed in *DEX:IPT* roots. C-NL conditions elevated *RGA1* and *2* transcription in *DEX:CKX* apices to the levels of the other genotypes. Significant elevation of *RGA1* transcription was found in leaves of WT and *DEX:IPT* plants under C-LL. C-D diminished the transcription of *RGA1* and *2* in all tissues of all genotypes, except roots of the *DEX:CKX* transformant, which maintained high transcription even in the dark. *RGL3* exhibited up-regulation under all stress conditions in apices of all genotypes (except in *DEX:IPT* under C-D). In leaves, significant elevation was observed under C-D in WT and *DEX:IPT* and in the latter genotype also under C-LL.

##### Strigolactones

Transcription of strigolactone receptor *MAX2* (*more axillary branches 2*) exhibited up-regulation under C-NL and C-LL in apices of all genotypes. Its transcription was also enhanced in leaves of *DEX:IPT* under all cold treatments (because of low basal transcription under control conditions). In roots, a sharp *MAX2* down-regulation was observed under C-NL. Strigolactone co-receptor *D14* (*dwarf 14*) transcription was also diminished in *DEX:IPT* under control conditions. It was increased under C-LL only in WT apices and leaves and, to a lesser extent, in *DEX:IPT* leaves. Transcription of strigolactone repressor *SMXL6* (*SMAX1-like 6*) was high under C-LL in leaves of *DEX:IPT* plants and in roots of all genotypes. C-D conditions generally down-regulated *SMXL6* transcription. As *MAX2* may function also as a karrikin receptor, transcription of karrikin co-receptor *KAI2* (*karrikin insensitive 2*) and karrikin repressor *SMAX1* (*suppressor of MAX2 1*) was also followed. Significant up-regulation of *KAI2* was found in apices and leaves of *DEX:IPT* plants exposed to C-NL and C-LL. By contrast, all stress treatments suppressed its transcription in roots, especially under C-D. *SMAX1* had very low transcript levels in apices of the C-D-treated plants. Moderate elevation was found under C-LL in WT leaves and in *DEX:IPT* roots.

#### Transcriptome Changes in Summary

Independent component analyses of transcriptomic changes (Figure 4B) revealed that the changes between cold stress under normal light (C-NL) and low light (C-LL) conditions were similar in apices, indicating the protective efforts of plants in maintaining growth processes in this tissue. By contrast, in leaves, the responses to both cold treatments under light clearly separated, especially in the case of WT. In the case of *DEX:CKX* plants, the changes in gene transcription were similar regardless of conditions. Under cold stress the transcriptomic changes in roots were distinct from those of control plants; however, the response under C-LL still differed from the other two stress treatments. The only exception was the *DEX:IPT* transformant, which resembled C-NL and C-D plants in showing distinct responses under control conditions in comparison with WT and *DEX:CKX* plants. As in the case of phytohormones, all genotypes in all tissues reacted similarly to cold in the dark (C-D).

### Proteome

#### Proteome Cold Stress Response in *Arabidopsis*

Analysis of C-NL conditions on WT revealed an accumulation of 202 and depletion of 35 proteins. The set of cold-induced proteins contained 41 well-known stress-responsive proteins, including enzymes of the ABA-biosynthetic pathway: zeaxanthin epoxidase (AT5G67030) and phytoene desaturase (AT4G14210); reactive oxygen species (ROS) metabolism (e.g., peroxidase PRXR1, AT4G21960); and chloroplastic enzymes of carbohydrate metabolism, e.g., phosphoglucomutase (AT5G51820), α-glucan water dikinase 1 (AT1G10760), or chalcone synthase (AT5G13930).

Low light intensity and darkness significantly altered the WT cold response at the proteome level. Of 237 cold-responsive proteins under C-NL, only 57 and 41 showed a similar trend under C-LL and C-D treatments, respectively. The functional enrichment analyses revealed mutual enrichment in the abiotic stress response, biosynthesis of secondary metabolites, amino acids, photosynthesis, and ribosomes. In contrast, carotenoid and flavonoid biosynthetic pathways were enriched only under C-NL, whereas a higher proportion of ROS metabolism, JA metabolism, and glucosinolate biosynthesis were found only under C-LL or C-D. Statistically significant changes were found for 173 proteins, and trends of most of these (94) were similar for C-LL and C-D treatments. The set of proteins specifically responsive under C-LL included enzymes of ROS metabolism, ascorbate peroxidase (AT4G35000, accumulated), and peroxidases (AT4G21960, AT4G33420, depleted); JA biosynthesis (allene oxide cyclase 2, AT3G25770, accumulated); and tryptophan and histidine biosynthesis (AT4G26900, AT1G07780, accumulated). The C-D-specific response included depletion of nine stress-responsive proteins, an increase in the ubiquitin–proteasome pathway (UBC8, AT5G41700; UBC35, AT1G78870; proteasome subunit AT1G79210), or protein required for vesicular transport (AT3G56190).

#### Modulation of CK Content Affected Proteome

The analysis of total leaf proteome allowed us to identify a total of over 20,000 peptides, representing more than 3,800 proteins and 2,212 protein families. DEX-induced expression of *ipt* and *HvCKX2* under control conditions resulted in a significant change of 232 proteins. A CK-dependent protein accumulation was found for 31 proteins, including mitochondrial chaperone ClpB4 (casein lytic proteinase B4, AT2G25140, negative response to CK); lipid transfer protein LPT5 (AT3G51600, negative); chloroplastic hydroxyacyl-glutathione hydrolase 2 (AT1G06130, negative); and alcohol dehydrogenase (AT1G77120, negative). A similar response of both transformants (*DEX:IPT* and *DEX:CKX*) was found for 24 proteins, including EIN2 interacting protein AT4G24800 (accumulated); a stress-induced protein KIN2 (kinase 2, AT5G15970, accumulated); amidase 1 (AT1G08980, depleted); and a splicing factor subunit AT1G14650 (depleted).

The functional enrichment analysis showed that of 112 proteins differentially abundant only in the *DEX:IPT* genotype, the most numerous categories were enzymes of amino acid biosynthesis (10 proteins), citrate cycle (6 enzymes), and ribosomal proteins (12 proteins). A notably significant depletion was found for a protein of thermotolerance TIL1 (temperature-induced lipocalin 1, AT5G58070), and enzymes spermidine synthase (AT1G70310) and arginase (AT4G08870), indicating a decrease in polyamine biosynthesis. Important proteins accumulated in the *DEX:IPT* transformant were phospholipase D (AT3G15730), MAP-kinase 4 (AT4G01370), and a mitochondrial protein prohibitin-3 (AT5G40770) that is required for the ethylene-mediated signaling (Christians and Larsen, 2007).

The *DEX:CKX* line-specific changes were only found for 65 proteins: emphasizing cold-regulated protein (AT2G42540, depleted); JA biosynthetic enzyme 12-oxophytodienoate reductase (AT2G06050, accumulated); sulfite oxidase (AT3G01910, accumulated); and histone H4 (AT5G59970, accumulated).

#### Cold Stress Response of Proteome Under Optimal Light Intensity Was Similar Among Genotypes

**FIGURE 7:**
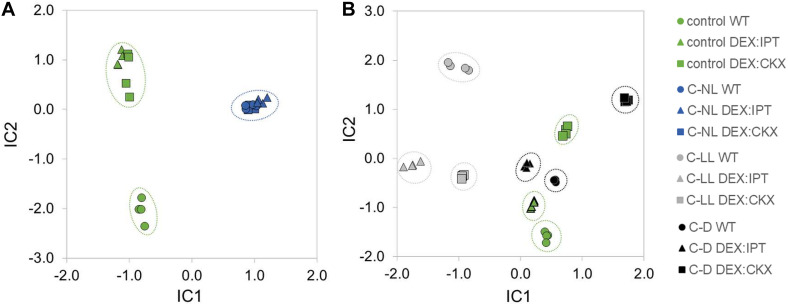
The independent component analysis (ICA) of proteome profiles in leaves of WT, *DEX:IPT*, and *DEX:CKX* plants. **(A)** Cold stress response at standard light intensity (C-NL) masks the effect of altered cytokinin pool. Results represent the quantitative analysis of 2,067 proteins in four replicates. **(B)** The response to cold stress under low light (C-LL) and dark (C-D) conditions in comparison with plants grown under control conditions. The ICA separates light intensity and cytokinin effects in the IC1 and IC2 axes, respectively. The results represent the quantitative analysis of 1,950 proteins in four replicates.

When the cold-responsive proteins were characterized in plants with a modulated CK pool, some of the observed changes were not statistically significant within the set of replicates, but of 237 cold-responsive proteins, 178 and 180 proteins showed a similar trend to that seen in the leaves of activated *DEX:IPT* and *DEX:CKX* plants, respectively. In accord with that observation, only 38 proteins that showed no cold response in WT were significantly changed in *DEX:IPT* and *DEX:CKX* plants under C-NL stress. The high similarity between plants with a modulated CK pool and WT plants under C-NL is demonstrated by ICA (Figure 7). The following enzymes were significantly accumulated in *DEX:IPT* and *DEX:CKX* plants under cold stress: FtsH protease (AT1G50250); tryptophan-tRNA ligase (AT3G04600); potassium/sodium channel protein (AT1G51100); and enzyme releasing ABA from its conjugate BGLU18 (β-glucosidase 18, AT1G52400).

Proteins accumulated specifically in leaves of cold-stressed *DEX:IPT* plants included glutathione S-transferase L3 (AT5G02790) and peptidyl-prolyl *cis-trans* isomerase CYP19-4 (AT2G29960). Photoreceptor phototropin-2 (AT5G58140) was significantly depleted, in contrast to control conditions, when it was in *DEX:IPT* plants substantially higher than in WT.

The set of differentially abundant proteins in cold-stressed *DEX:CKX* plants included a protein involved in programmed cell death NUDT7 (nudix hydrolase homolog 7, AT4G12720), a positive regulator of abiotic stress tolerance and ABA target lipoxygenase PLAT1 (AT4G39730), and a thioredoxin-family protein (AT4G29670).

#### The Impact of Low Light Intensity on Proteome During Cold Stress Response

In total, 188 proteins that were not found to be cold-responsive in WT in our test conditions were significantly changed in the *DEX:IPT* plants. The comparison of the sets from C-LL and C-D showed a higher overlap of the cold-accumulated proteins, with ca 27 and 16% shared accumulated and depleted proteins, respectively. The mutual C-LL and C-D accumulated proteins in *DEX:IPT* included ribosomal proteins; hydroxyacyl-glutathione hydrolase (AT1G06130); an enzyme with a putative role in sucrose mobilization (FRUCT4, fructosidase 4, AT1G12240); NADPH-dependent thioredoxin reductase (NTRB, AT4G35460); pectin acetylesterase 11 (AT5G45280); and methyltransferase FIB2 (fibrillarin 2, AT4G25630). In *DEX:IPT* plants, the set of depleted proteins common to both C-LL and C-D treatments contained phospholipase D (AT3G15730) and glucan endo-1,3-β-glucosidase. C-LL alone resulted in accumulation of citric acid cycle enzymes (AT5G40650, AT1G54220); 14-3-3 protein (AT2G42590); actin 1 (AT2G37620); and magnesium-chelatase subunit (AT5G45930). The set of *DEX:IPT* proteins depleted under C-LL conditions included NAD-dependent malic enzyme (AT4G00570); a negative regulator of ABA signaling nodulin-related protein 1 (AT2G03440); a subunit of DNA-directed RNA polymerase (ATCG00740); and chaperones and ribosomal proteins. Dark-specific (C-D) proteome changes in the *DEX:IPT* transformant included an accumulation of histone H2B.11 (AT5G59910); enzymes of amino acid biosynthesis and glutathione metabolism; an increase in HSP 90-6 (AT3G07770); and protein calnexin (AT5G61790) that may participate in glycoprotein assembly. Three citric acid cycle components were depleted in *DEX:IPT* under C-D: a decrease was found in the case of HSP70-14 (AT1G79930) and its interacting protein HIP1 (AT4G22670); SGT1 protein (AT4G11260) required for degradation of auxin-responsive proteins; plastid lipid-associated proteins (AT3G26070, AT3G58010, AT3G23400); proteasome subunits; and ribosomal proteins.

In total, 154 proteins that were not found to be cold-responsive in WT under C-LL or C-D were significantly changed in those plants overexpressing *HvCKX2*. The comparison of the sets of C-LL- and C-D-affected proteins in *DEX:CKX* showed a limited overlap of 16 and 14 accumulated and depleted proteins, respectively. These included auxin biosynthetic enzyme AMI1 (amidase 1, AT1G08980, accumulated); cold-regulated protein COR15A (AT2G42540, accumulated); importin subunit (AT1G09270, accumulated); ABA receptor PYL1 (AT5G46790, depleted); and growth-limiting enzyme of guanine biosynthesis (AT1G16350, depleted). The positive response in the *DEX:CKX* transformant under C-LL was found in the case of ribosomal proteins; enzymes of carbohydrate metabolism; mannose-binding lectin (AT3G16470); chalcone synthase (AT5G13930); ABA biosynthesis enzyme zeaxanthin epoxidase (AT5G67030); histone H3-like (AT5G65350); malate dehydrogenase (AT5G43330); and HHL1 (hypersensitive to high light 1, AT1G67700). Negative responses of *DEX:CKX* plants under C-LL were found for two enzymes involved in aromatic amino acid biosynthesis (AT5G38530, AT1G48850); receptor AT4G23170 similar to NPR1 (positive regulator of SA signaling); two enzymes of sulfur metabolism (AT1G79230, AT3G01910); glutathione-*S*-transferase (AT1G78380); and thioredoxin reductase 2 (AT2G17420). The cold-stress-specific response of *DEX:CKX* plants under C-D included an accumulation of lipid peroxidation protectant lipocalin (AT3G47860); enzymes of fatty acid biosynthesis (AT1G65290, AT5G10160); HSP70-15 protein (AT1G79920); actin-depolymerizing factor 3 (AT5G59880); and tubulin (AT5G19780). In contrast, tubulin (AT1G20010) was depleted under C-D; a decrease was found also for multiple ribosomal proteins, enzymes of porphyrin and chlorophyll metabolism (AT4G03205, AT4G03205), thioredoxin X (AT1G50320), and a protein involved in multivesicular body formation CHMP1A (charged multivesicular body protein/chromatin modifying protein 1A, AT1G73030).

#### Proteome Changes in Summary

The modulation of CK pool had a significant impact on cold stress-responsive proteins in leaves of all tested genotypes. The ICA separated light intensity and CK effects in the IC1 and IC2, respectively (Figure 7). WT plants showed the most distinct responses to stresses at the proteome level. In contrast to hormonome and transcription analyses, proteome data clustered all genotypes grown under C-NL stress together. More differences were found under C-LL conditions, especially when transformants were compared with WT. C-D and control conditions resembled each other (as in the case of hormonome and transcriptome). C-D had a much higher impact on *DEX:CKX* plants than on the other two genotypes; however, its proteome was likewise shifted also under control conditions.

## Discussion

### The Impact of Light Intensity on Cold Stress Responses

The first goal of this study was to investigate the effect of different light intensities on cold stress responses in *A. thaliana* WT. The impact of cold stress under normal light (C-NL, 150 μmol m^–2^ s^–1^), low light (C-LL, 20 μmol m^–2^ s^–1^), and dark (C-D, 0 μmol m^–2^ s^–1^) was evaluated after 1-week exposure to reveal long-term stress responses. The comparison of phenotypes is shown in Supplementary Figure 2. At standard light intensity (C-NL), apices showed preferential protection against cold stress, exhibiting milder reaction in comparison with leaves. At low light intensity (C-LL), similar stress responses were observed in these tissues. Dark in combination with cold stress (C-D) arrested plant growth.

#### Cold Stress Under Normal Light Conditions

Cold stress under normal light conditions resulted in significant membrane damage and moderate photosynthesis suppression, but plants were simultaneously stimulated to form protective compounds (e.g., flavonoids and chaperones), including antioxidant enzymes (peroxidases) (Figure 1, Table 2). The observed negative effect of cold stress on photosynthesis agrees with reports of Janda et al. (2014) and Prinzenberg et al. (2020). This cold stress response was associated with significant transcriptome changes (Figure 6) including the transcription of stress-related genes *RD29A*, *COR47*, and *CBF1–3*. The transcription of *CRF3* and *4* was induced by C-NL in above-ground tissues but suppressed in roots, which is in accordance with Zwack et al. (2016), who reported their cold induction and positive function in freezing tolerance. The transcription of *PIFs* was enhanced in leaves (but diminished in roots), which conforms with their function in cold stress responses as a part of CBFs-PIF3-phyB regulatory module (Jiang et al., 2020). Under cold stress, CBFs interact with PIF3 to facilitate the degradation of PIF1, PIF4, and PIF5, which repress transcription of COR genes (Xu and Deng, 2020).

Prolonged cold was associated with down-regulation of bZIP transcription factor *HY5*, especially in roots. This is in apparent contradiction with the fact that transcription of *HY5* is both cold- and light-inducible. However, the reason may be *HY5* dynamics, as its maximum has been reported to occur 3 h after cold stress initiation (Catala et al., 2011). HY5 is a convergence point between light and hormone signaling pathways (Lau and Deng, 2010). It suppresses auxin signaling via activation of its negative regulators [e.g., IAA7 (indole-3-acetic acid inducible 7)], promotes ABA signaling by interaction with ABI5 (ABA insensitive 5), and stimulates GA deactivation (Lau and Deng, 2010). CKs enhance the stability of HY5 protein by diminishing its degradation by COP1 (Vandenbussche et al., 2007), while GAs have the opposite effect (Alabadi et al., 2008).

Cold stress led to significant changes in hormone pools (see Figures 2, 3, 5, 6). The content of ABA increased in all tissues, as well as *NCED3* transcription and abundance of ABA-biosynthetic enzymes in leaves. The ABA sensitivity (estimated by *PYL6* transcription) was elevated in apices and leaves. Cold stress diminished tZ and tZR levels in WT apices, the degree depending on light intensity (C-NL > C-LL > C-D). C-NL strongly stimulated *CKX* transcription as well as the enzyme activity in leaves, indicating a high rate of CK degradation. Nevertheless, *CKX5* transcription was diminished in apices and roots, which could indicate that CK down-regulation in these tissues was caused by other CKX isozymes, such as CKX4 or 6 (Wang et al., 2020), or by a low rate of CK biosynthesis. The level of iP precursor [*N*^6^-(Δ^2^-isopentenyl)adenosine monophosphate] was increased in leaves, which may suggest the replacement of highly active tZ with less active iP (Spichal et al., 2004). Decrease of transcription of type-A ARRs suggests that suppression of CK signal transduction was eliminated after prolonged stress, probably to allow plant acclimation, associated with CK content increase (Kosova et al., 2012). This is in accordance with Jeon et al. (2010), who found that overexpression of *ARR7* resulted in reduced cold tolerance. Decrease of CKs was accompanied by inactivation of auxins to reversible storage forms, which were found up-regulated in all tissues (Supplementary Table 2). C-NL seems to be associated with down-regulation of GAs in apices, as indicated by up-regulated transcript levels of GA repressor *RGL3*. Up-regulation of strigolactone receptor *MAX2* in apices suggests suppression of primordia development. Inhibition of branching or tillering is one of the most distinct strigolactone physiological functions (Waldie et al., 2014). Down-regulation of *MAX2* transcription in roots may be connected with inhibition of root growth in cold stress. Strigolactones have been reported to enhance tolerance to several abiotic stresses, e.g., drought and salinity (Mostofa et al., 2018); thus, it is possible that they also play a role in cold stress responses. Moreover, Koltai et al. (2011) reported a positive correlation between light intensity and strigolactone levels in tomato roots. The parallel karrikin pathway was suppressed by cold in general. This is in apparent contradiction with the studies of Zhao et al. (2012) and Zheng et al. (2015), who suggested that karrikin has a role in cold or freezing stress responses in *Chorispora bungeana* and *Camellia sinensis*, respectively. However, both studies determined transcription profiles at the early phase of cold stress (after 4–8 h and 24 h, respectively). Karrikin has been studied predominantly in relation to germination promotion (e.g., Bunsick et al., 2020).

#### Cold Stress Under Low Light Conditions

Cold stress under low light conditions caused substantial membrane damage (Figure 1). The response to cold stress under low light differed significantly from that under normal light at the level of transcriptome, hormonome, and proteome (Figures 4, 7). Cold treatment at low light intensity has been found to result in much lower acquired freezing tolerance than at normal light conditions (Szalai et al., 2009; Janda et al., 2014). Nevertheless, transcription of the stress marker genes *RD29A* and *COR47* was stimulated much more than in the case of C-NL (*RD29A* in roots, *COR47* in all tissues; Figure 6). On the other hand, *CBF1–3* genes were less up-regulated under C-LL than under C-NL. Elevation of *PIF* transcription in leaves under C-LL and C-NL was similar.

Plants exposed to C-LL had ABA and jasmonate levels up-regulated in all tissues, as well as SA content in roots (Figure 3). CK content under C-LL was lower than under C-NL (Figure 2 and Supplementary Table 2). C-LL was the only treatment that stimulated production of the less active CK cZ (and its riboside), which has been connected with stress responses, preserving certain CK functions under conditions of suppressed growth (Gajdosova et al., 2011). CK signal transduction during C-LL, estimated via *ARR10* transcription, was maintained or slightly up-regulated in all tissues (Figure 6). Significant up-regulation of the transcription of negative regulators (*ARR4*, *5*, and *7*) was, however, detected in leaves. C-LL was associated with only minor changes in IAA content (Figure 3) compared with control, which agrees with a reported positive auxin function at skotomorphogenesis (Ballare and Pierik, 2017). Simultaneously, storage conjugate glucose ester of IAA was formed in roots (Supplementary Table 2). Prolonged C-LL stimulated transcription of GA repressors *RGA1* in leaves and, to a minor extent, of *RGL3* in apices (Figure 6). Up-regulation of strigolactone co-receptor *D14* in apices and leaves (as in the case of C-NL), together with a decrease of the strigolactone repressor *SMXL6* transcript in roots, seems to indicate that strigolactones participate in cold stress responses at low light intensity. These data agree with Xie et al. (2020) who found that, in shade, strigolactones stimulate SMXL6/7/8 degradation, promoting the expression of transcription factor *BRC1*, which inhibits bud outgrowth.

**FIGURE 8:**
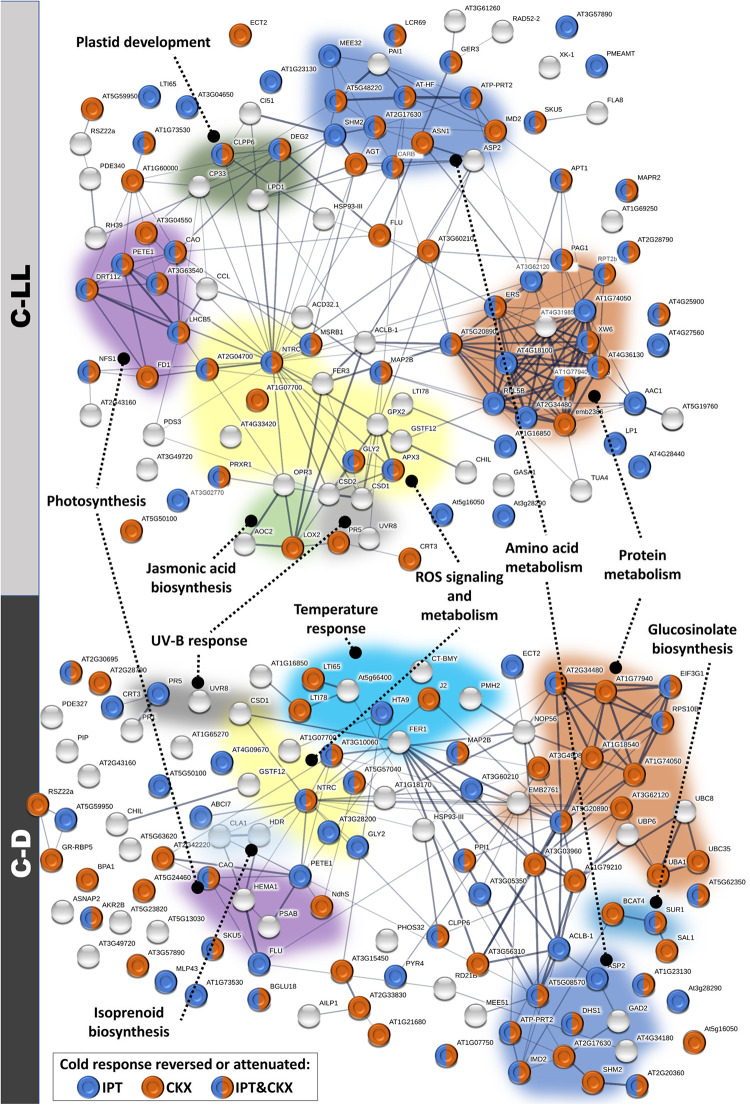
Modulation of cold-responsive proteins in plants with an altered cytokinin pool. Interactions and functional clusters of identified cold-responsive proteins (compared to control, 20°C, 150 μmol m^–2^ s^–1^; *p* < 0.05) were highlighted by String (Szklarczyk et al., 2019). The line thickness indicates the strength of data support, and the minimum required interaction score is 0.4 (medium confidence). See proteomic data in ProteomeXchange for details.

Cold stress under low light resulted in increased abundance of proteins associated with the abiotic stress response, as well as the biosynthesis of secondary metabolites such as glucosinolates (Figure 8). The low light-specific cold-responsive (C-LL) proteins included enzymes of ROS metabolism, e.g., ascorbate peroxidase.

#### Cold Stress Under Dark Conditions Caused Plant Growth Arrest

Absence of light during cold stress (C-D) partially diminished the stress impact on membrane peroxidation in comparison with the other cold treatments, C-NL or C-LL (Figure 1). In spite of the relatively low level of oxidative damage, C-D conditions led to the most significant reduction in both the maximal and actual quantum efficiency values (Table 2), indicating the presence of a secondary, dark-induced stress. However, it must also be mentioned that the lowest *F*_*v*_/*F*_*m*_ values were still around 0.8, indicating that the stress was indeed mild, and at least the photosynthetic machinery was in a relatively good physiological state. Transcription of the stress-related gene *COR47* was only moderately up-regulated in apices and leaves, while *RD29A* was elevated considerably less than in the light treatments (Figure 6). *CBF1* (in contrast to *CBF2* and especially *CBF3*) was up-regulated to a similar extent as in the light conditions, indicating its dependence on temperature rather than light intensity. Transcription of *CRF4* and especially of *CRF3* was low in comparison with other cold treatments. *HY5* transcription was strongly diminished in the dark, which is in agreement with its association with photomorphogenesis (Lau and Deng, 2010). *PIF4* was highly stimulated in roots of C-D-treated plants, which could be related to its role in skotomorphogenesis (Ballare and Pierik, 2017).

Abscisic acid production was stimulated only in above-ground tissues, but it was not accompanied with the promotion of the levels of deactivation metabolites or transcription of *NCED3* and *PYL6* (Figures 3, 6 and Supplementary Table 2). On the other hand, jasmonates were down-regulated in all tissues. C-D resulted in very low levels of all CKs, their metabolites, as well as low *IPT3* and *ARR10* transcription, which consequently led to low transcription of type-A *ARRs* and CKX activity (Figures 2, 5, 6 and Supplementary Table 2). CK suppression is in accordance with negative function of CKs in skotomorphogenesis, described by Ballare and Pierik (2017). Nevertheless, transcription of *IPT5*, the enzyme predominant in roots, was substantially elevated in leaves and roots of dark-treated plants. This accords with the finding that a functional CK signaling pathway is necessary also in the dark in order to maintain transcripts of some plastid genes, to enable plants to keep their photosynthetic apparatus ready in case of environmental changes (Doroshenko et al., 2016).

Auxins were degraded to irreversible conjugates (Supplementary Table 2). C-D treatment did not significantly affect the content of precursor GA_19_, but transcription of GA repressors *RGA1* and *2* was down-regulated (Figures 3, 6). This may indicate that plants in the dark had high level of GAs, which could be connected with their role in skotomorphogenesis (Ballare and Pierik, 2017). Repression of the strigolactone signaling pathway via SMXL6 was inhibited, but the karrikin pathway was diminished most in all treatments.

The evidence of attenuated metabolism under C-D conditions was also evident from proteome analysis showing the up-regulation of the ubiquitin–proteasome pathway and vesicular transport (Figure 8).

### The Effect of Modulated CK Levels

#### Modulation of CK Levels Changed Hormone Pools as Well as Basal Metabolism in Plants

To investigate the effect of modified content of CKs on cold stress responses, transformants with increased CK biosynthesis (*DEX:IPT*) or degradation (*DEX:CKX*) were used. In order to avoid changes in plant morphology, expression of introduced genes was activated by DEX, 24 h before stress initiation. The effect of DEX on plants was checked at the level of membrane damage, chlorophyll fluorescence, and CKX activity (Figures 1, 5 and Table 2). DEX had only a mild (but significant) impact on all abovementioned parameters under control conditions, being of negligible importance in comparison with the intensity of changes imposed by modulated CK content or the cold stress.

Elevated endogenous CK levels caused by the introduced *ipt* gene from *A. tumefaciens* (*DEX:IPT* plants) substantially changed metabolism and signaling processes in plants. The transcription of the crucial CK biosynthetic enzyme *IPT3* was strongly suppressed, while CKX expression and activity as well as CK deactivation via CK *N-* and *O-*glucosylation pathways were elevated in order to down-regulate high CK levels and re-establish (at least partially) CK homeostasis (Figures 2, 5, 6 and Supplementary Table 2). CK signaling in apices and leaves was also regulated by stimulation of the transcription of negative response regulators (*ARR4*, *5*, and *7*).

Due to the hormone cross-talk, levels of IAA, ABA, SA, and JA were also moderately elevated (Figure 3). Auxin up-regulation in apices may support, together with CKs, meristem growth. The level of ABA, as well as *NCED3* transcription in apices (Figures 3, 6), was high in *DEX:IPT* compared with WT, which could indicate the maintenance of an optimal ABA:CK ratio, as described in previous studies (e.g., Dobra et al., 2015; Skalak et al., 2016; Pavlu et al., 2018). However, ABA perception via PYL6 was down-regulated under control conditions. SA levels correlated well with CK content, as the *DEX:IPT* transformant had a higher basal level of SA (Figures 2, 3). Signaling pathways of both hormones can interact, forming a complex between the type-B response regulator ARR2 and transcription factor TGA3, which increases plant tolerance to pathogen attack (Choi et al., 2010). Moderate JA elevation agrees with the reported positive CK effect on JA levels upon wounding (Sano et al., 1996). *DEX:IPT* plants showed low *MAX2* and *D14* transcription compared with WT, which may reflect antagonistic hormone relationship, as CKs and strigolactones regulate by opposing a number of physiological processes, e.g., bud or root growth (Dun et al., 2012).

Elevated content of CKs boosted synthesis of amino acids, sugar metabolism, and transcription (up-regulation of ribosomal proteins). These plants also contained more phototropin-2, which may reflect the CK link with light signaling (Werner and Schmulling, 2009). In summary, improved growth, but diminished transcription of stress-related genes *RD29A* and *COR47*, and translation of stress-related proteins in *DEX:IPT* plants support the hypothesis that plants with higher CK levels are delayed in sensing stress (Havlova et al., 2008).

Decreased endogenous CK levels caused by the introduced *HvCKX2* gene from *H. vulgare* (*DEX:CKX* plants) suppressed growth and increased the level of stress-related compounds under control conditions. CKX activity was moderately elevated, but this increase did not correlate with high *HvCKX2* transcription, probably due to attenuation of CKX protein function or its rapid degradation (Figures 2, 5). Moreover, this genotype exhibited down-regulated CK deactivation through CK glucosylation and transcription of endogenous CKXs (e.g., *CKX5*), while transcription of CK biosynthetic enzymes (*IPTs*) and positive response regulators (*ARR10*) was significantly enhanced (Figure 6 and Supplementary Table 2). Negative regulation of CK signaling via type-A ARRs (*ARR4*, *5*, and *7*) was diminished. A high content of tZRMP (precursor of tZ) in roots was accompanied by synthesis of cZR, which is readily convertible to the low active cZ, which could in turn be set aside as the substrate of the HvCKX2 enzyme.

*DEX:CKX* exhibited a generally lower transcription of *CRF3* and *4*, which corresponds with their lower cold tolerance as indicated by higher MDA levels after cold treatments (Figures 1, 6). Low CK levels down-regulated the production of auxin IAA, which might contribute to plant growth suppression (Figure 3). Correlation of SA content with CK levels was also observed in this genotype. By contrast, *NCED3* transcription and ABA content were stimulated in apices and leaves (Figures 3, 6). Subsequently, *RD29A* transcription and translation of stress-related proteins (including a JA biosynthetic enzyme) were enhanced. *DEX:CKX* plants had low levels of GA_19_ precursor indicating low GA content leading to growth retardation (Ross et al., 2001).

Independent component analyses of phytohormone pools (Figure 4A) showed distinct responses of *DEX:IPT* plants, especially in apices and leaves. *DEX:CKX* plants differed from WT under control conditions significantly in roots. ICA of transcriptional data (Figure 4B) showed the most distinct differences among genotypes in roots. A clear separation of the genotypes at the proteome level (Figure 7) reflected the higher distance of *DEX:CKX* in comparison with WT and *DEX:IPT*.

#### Elevated CK Content Up-Regulated Stress Tolerance

The transcription of the *ipt* transgene was generally stable regardless of cold stress (Figure 6). Only the C-D treatment partially down-regulated *ipt* transcription. The huge amount of CKs was diminished by cold treatments, but the levels in *DEX:IPT* apices were, under C-NL and C-LL, still higher than in WT under control conditions (Figure 2). The elevated CK content in *DEX:IPT* plants maintained up-regulated negative feedback on CK signaling in apices and leaves via up-regulation of transcription of type-A response regulators in all stress treatments (Figure 6). The positive effect on IAA levels in apices was also preserved during cold stress treatments, as it was under control conditions (Figure 3). ABA levels in *DEX:IPT* plants were high under cold stress, especially in apices and roots under C-LL. The ABA content also seems to correlate with a higher abundance of enzyme releasing ABA from storage conjugates under C-NL. ABA signaling via PYL6 was enhanced in apices and leaves under both C-NL and C-LL, and SA elevation was partially maintained under the same conditions in apices. In roots, high SA levels were observed in all genotypes. In contrast, C-NL and C-LL stressed plants suppressed the strigolactone signaling pathway in *DEX:IPT* plants. This finding may reflect the fact that strigolactones exhibit antagonistic relationships with CKs (Dun et al., 2012). The karrikin pathway seemed to be enhanced.

Despite low basal transcription of *RD29A* under control conditions, *DEX:IPT* plants showed its highest elevation under C-NL stress, which may contribute to the diminished negative impact of cold stresses on the level of membrane peroxidation in *DEX:IPT* plants (Figures 1, 6). A further contribution to this effect might arise from changes in leaf proteome, since C-NL stress stimulated in *DEX:IPT* plant proteins associated with correct folding (CYP19-4) as well as the production of protective compounds (glutathione *S*-transferase). C-LL conditions regulated transcription as well as saccharide metabolism (Figure 8). *DEX:IPT* plants exposed to C-LL showed moderately elevated transcription of *HY5* in comparison with other genotypes (Figure 6), which might arise from a synergy between light and CK signals. Despite the fact that C-D conditions elicited similar responses in both WT and the transformants, *DEX:IPT* plants still exhibited a higher transcription of negative CK signaling regulators as well as *RD29A*, *COR47*, and *CBF1* in leaves. Under C-D, *DEX:IPT* plants were able to maintain the higher translation of proteins involved in amino acid biosynthesis, transcription activity (histone accumulation, ribosomal proteins), protein folding (HSPs), and glycoprotein assembly (Figure 8).

Our data are in accord with reported positive effects of elevated CK contents on cold stress tolerance, e.g., in the callus of tall fescue transformed with *ipt* under maize ubiquitin promoter (Hu et al., 2005), or in *COR15a:ipt* sugarcane transformant (Belintani et al., 2012). It seems that despite lower preparedness of *DEX:IPT* plants to stress, they are able to stimulate stress-defense mechanisms protecting against cold stress effectively. Nevertheless, the impact of cold stress on CK levels as well as on CKX activity in the transformants indicate that cold stress is a predominant regulatory factor in comparison with transgene activation (see ICA at Figures 4, 7).

Independent component analyses showed very distinct cold stress responses at different light intensities (Figures 4, 7). ICA of hormone pools showed a clear separation of *DEX:IPT* plants between C-NL and C-LL especially in apices, the distance in the leaves being lower, while a close proximity was observed in roots. ICA of transcription data showed clear separation between C-NL and C-LL in leaves and roots. Proteome ICA revealed quite close proximity under C-NL, while under C-LL and C-D, distinct differences were observed among genotypes as well as treatments.

#### Low CK Content Had a Negative Impact on Cold Stress Tolerance of Plants

Because *DEX:CKX* plants exhibited intensive suppression of the transgene activity under all cold stresses, especially in apices of plants exposed to C-LL (Figure 6), low CK content seems to be a serious disadvantage during cold stress responses. In comparison with control conditions, tZ and iP were significantly up-regulated under C-NL in *DEX:CKX* apices and roots (Figure 2). The cZRMP in roots of this genotype reached a higher level than in stressed WT. Even up-regulation of storage metabolites CK *O*-glucosides was detected (Supplementary Table 2). The overexpression of *HvCKX2* led to stimulation of the transcription of *IPT3* gene in leaves and roots (Figure 6). C-LL stressed plants also had diminished transcription of negative regulators of CK signaling (*ARR4*, *5*, and *7*) in leaves. Despite the intensive regulation of endogenous CKs, lower CK content strengthened the negative impact of cold stress on membrane damage (Figure 1). This finding may also be due to the fact that transcription of *RD29A* in apices and leaves was not as high as it was in the other genotypes (Figure 6). Also, C-NL and C-LL responses were associated with lower up-regulation of *CBFs*, suggesting a weaker cold stress reaction by this genotype.

The C-NL treatment diminished transcription of ABA biosynthetic gene *NCED3* in leaves of *DEX:CKX* plants; however, the enzyme releasing ABA from storage conjugates was up-regulated; thus, ABA content remained high, comparable to values in other genotypes (Figures 3, 6). Stress-related proteins connected with programmed cell death, thioredoxin, or ABA targeting lipoxygenase were exclusively up-regulated in *DEX:CKX* plants exposed to C-NL. This cold treatment also led to the increase of transcription of DELLA proteins in apices. Both light treatments (C-NL and C-LL) were associated with the elevation of SA content in roots to a level comparable with other genotypes.

The enhanced abundance of auxin biosynthetic enzyme amidase 1 under C-LL and C-D conditions may be responsible for IAA elevation in apices and roots under C-LL, and in apices only, under C-D (Figures 3, 8). Up-regulation of ABA biosynthetic enzymes (NCED3, zeaxanthin epoxidase) in leaves and roots of *DEX:CKX* plants under C-LL may be the cause of relatively high ABA levels in all tissues, comparable with WT (Figures 3, 6, and 8). C-LL also led to an increase of SA comparable to that in WT. Jasmonates were maintained at high levels in roots during C-D stress, higher than in the other genotypes. *DEX:CKX* plants had diminished levels of glutathione-S-transferase and thioredoxin. In leaves, high levels of proteins associated with cytoskeleton re-organization, lipid peroxidation protector (lipocalin), and fatty acid synthesis were found. Surprisingly, HHL1 protein, which is involved in protection against high light stress, was up-regulated under low light conditions (C-LL) in leaves of *DEX:CKX* plants. C-D treatment had a slightly more negative impact on photosynthetic capacity (*F*_*v*_/*F*_*m*_) than in WT, which was in agreement with decrease in proteins of chlorophyll synthesis and translation (Figure 8 and Table 1). These results correlate well with reported CK functions in the synthesis and stabilization of chlorophyll (Werner and Schmulling, 2009).

## Conclusion

Light intensity strongly affected the cold stress response. At low light intensity, the negative effects of cold on photosynthesis were diminished. A combination of dark and cold arrested the growth of plants regardless of genotype, resulting in a strong suppression of CK levels.

Cold stress strongly affected CK levels in transformants with high (*DEX:IPT*) or low (*DEX:CKX*) CK contents, considerably diminishing the effect of inserted genes. The influence was predominant in roots, the primary location of CK production. Nevertheless, modulation of CK levels still had a significant effect on cold stress responses. Although *DEX:IPT* plants under control conditions predominantly targeted available energy to growth, exhibiting only a low level of transcription of stress-related genes (especially in leaves and roots), they were able, upon cold stress, to acclimate well to unfavorable conditions. On the other hand, *DEX:CKX* plants exhibited better “stress adjustment” under control conditions, but their ability to acclimate in prolonged stress was worse.

## Data Availability Statement

The datasets presented in this study can be found in online repositories. The names of the repository/repositories and accession number(s) can be found below: https://www.ebi.ac.uk/pride/archive/projects/PXD020480.

## Author Contributions

RV designed the experiment. VK and SP prepared plant material. PD, AG, VK, and SP analyzed phytohormone contents. SP, BZ, and LH determined gene transcription. MČ and BB performed proteome analyses. VM determined CKX activity. TJ analyzed photosynthetic parameters. SP, RV, and MČ evaluated results and prepared the publication. All authors contributed to manuscript revision, and read and approved the submitted version.

## Conflict of Interest

The authors declare that the research was conducted in the absence of any commercial or financial relationships that could be construed as a potential conflict of interest.

## Footnotes

1 www.real-statistics.com

2 www.rapidminer.com

## Supplementary Material

Supplementary Figure 1 Shoot and root mass.

Supplementary Figure 2 Plant phenotypes at different experimental conditions. Proteomic data are available via ProteomeXchange (http://www.proteomexchange.org/) with identifier PXD020480. For the purpose of peer review, the data are accessible under the username reviewer41015@ebi.ac.uk and the password qFxVQj3A.

Supplementary Table 1 List of qPCR primers.

Supplementary Table 2 Phytohormones.

Supplementary Table 3 Transcriptomic data.

## Abbreviations

ABA: abscisic acid
C-D: cold and dark treatment
C-LL: cold and low light treatment
C-NL: cold and normal light treatment
CK: cytokinin
CKX: cytokinin oxidase/dehydrogenase
cZ: *cis*-zeatin
cZR: *cis*-zeatin riboside
cZRMP: *cis*-zeatin riboside monophosphate
DEX: dexamethasone
DMSO: dimethyl sulfoxide
*F*_*v*_/*F*_*m*_: maximum quantum yield of photosystem II
GA: gibberellin
IAA: indole-3-acetic acid
ICA: independent component analysis
iP: *N*^6^-(Δ ^2^-isopentenyl)adenine
IPT: isopentenyl transferase
JA: jasmonic acid
MDA: malondialdehyde
NPQ__*Lss*_: the steady-state non-photochemical quenching
QY__*Lss*_: the steady-state PSII quantum yield
ROS: reactive oxygen species
SA: salicylic acid
tZ: *trans*-zeatin
tZR: *trans*-zeatin riboside
tZRMP: *trans*-zeatin riboside monophosphate
WT: wild-type.
