# Supplementary material for: Light Regulates the Cytokinin-Dependent Cold Stress Responses in Arabidopsis
Source: Front Plant Sci. 2021 Feb 4;11:608711. doi: 10.3389/fpls.2020.608711 (PMC7889523; doi:10.3389/fpls.2020.608711)
Supplement: Supplementary Figure 1 — Shoot and root mass. [file Data_Sheet_1.PDF]

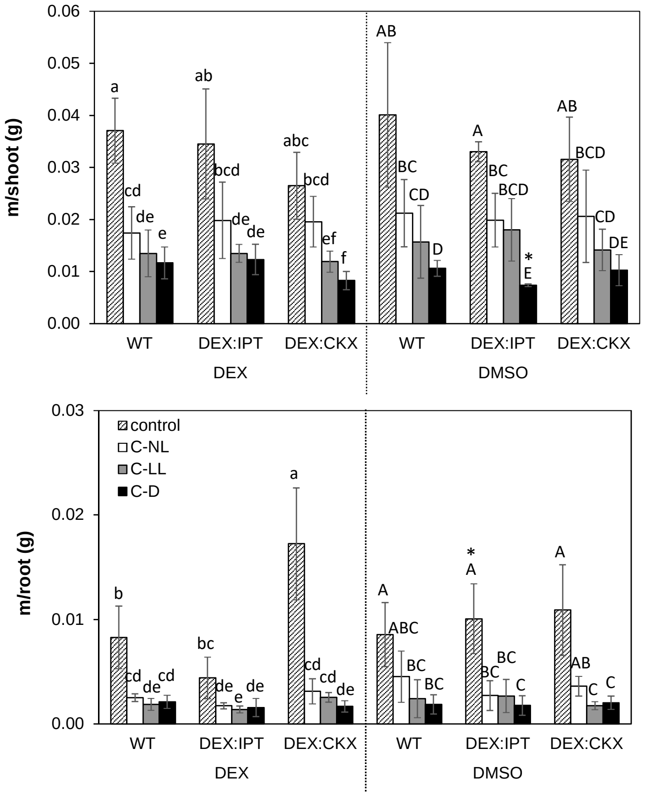

Figure S1: Mass of shoot and root per one plant of WT, DEX:IPT and DEX:CKX exposed to control conditions (150 µmol m $^{-2}$ s^{-1}, 20°C), cold under normal light (C-NL; 150 µmol m^{-2} s^{-1}, 5°C), cold under low light (C-LL; 20 µmol m $^{-2}$ s^{-1}, 5°C) and cold under dark (C-D; 0 µmol m $^{-2}$ s^{-1}, 5°C). Plants were either activated with dexamethasone (DEX; diluted in DMSO) or treated with DMSO only. Means ± SD are shown. Data for 1 plant were obtained from the total mass of ca 60 plants grown in two hydroponics vessels within one experiment divided by the number of collected plants. Three independent biological experiments were analyzed (n = 3). The differences between DEX and DMSO treatments within each experimental variant were evaluated by Student's t-test (significant differences at $p < 0.05$ are indicated with an asterisk). The comparison among all experimental variants within the DEX (lower-case letters) or DMSO (capital letters) treatments was evaluated by one-way ANOVA with Tukey's post-hoc test ($p < 0.05$).
