# Supplementary material for: Light Regulates the Cytokinin-Dependent Cold Stress Responses in Arabidopsis
Source: Front Plant Sci. 2021 Feb 4;11:608711. doi: 10.3389/fpls.2020.608711 (PMC7889523; doi:10.3389/fpls.2020.608711)
Supplement: Supplementary Figure 2 — Plant phenotypes at different experimental conditions. Proteomic data are available via ProteomeXchange (http://www.proteomexchange.org/) with identifier PXD020480. For the purpose of peer review, the data are accessible under the username reviewer41015@ebi.ac.uk and the password qFxVQj3A. [file Data_Sheet_2.PDF]

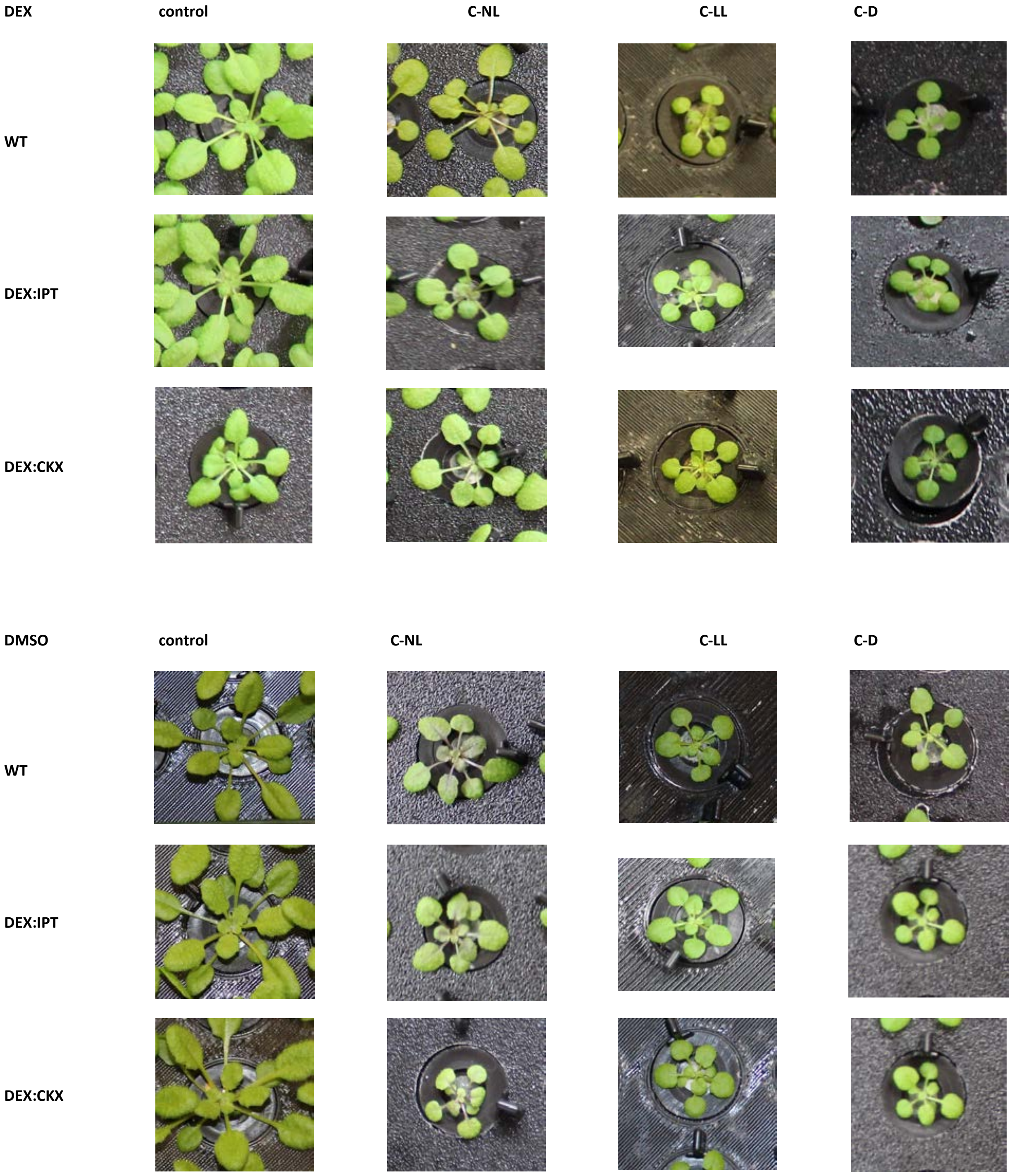

Figure S2: Phenotype of wild-type (WT) and transformants DEX:IPT and DEX:CKX exposed to control conditions (150 µmol m^{-2} s^{-1}, 20°C), cold under normal light (C-NL; 150 µmol m^{-2} s^{-1}, 5°C), cold under low light (C-LL; 20 µmol m^{-2} s^{-1}, 5°C) and cold under dark (C-D; 0 µmol m^{-2} s^{-1}, 5°C). Plants were either activated with dexamethasone (DEX; diluted in DMSO) or treated with DMSO only.
